# Feature importance feedback with Deep Q process in ensemble-based metaheuristic feature selection algorithms

**DOI:** 10.1038/s41598-024-53141-w

**Published:** 2024-02-05

**Authors:** Jhansi Lakshmi Potharlanka, Nirupama Bhat M

**Affiliations:** Department of Computer Science and Engineering, Vignan’s Foundation for Science Technology and Research, Guntur, 522213 India

**Keywords:** Computer science, Software

## Abstract

Feature selection is an indispensable aspect of modern machine learning, especially for high-dimensional datasets where overfitting and computational inefficiencies are common concerns. Traditional methods often employ either filter, wrapper, or embedded approaches, which have limitations in terms of robustness, computational load, or capability to capture complex interactions among features. Despite the utility of metaheuristic algorithms like Particle Swarm Optimization (PSO), Firefly Algorithm (FA), and Whale Optimization (WOA) in feature selection, there still exists a gap in efficiently incorporating feature importance feedback into these processes. This paper presents a novel approach that integrates the strengths of PSO, FA, and WOA algorithms into an ensemble model and further enhances its performance by incorporating a Deep Q-Learning framework for relevance feedbacks. The Deep Q-Learning module intelligently updates feature importance based on model performance, thereby fine-tuning the selection process iteratively. Our ensemble model demonstrates substantial gains in effectiveness over traditional and individual metaheuristic approaches. Specifically, the proposed model achieved a 9.5% higher precision, an 8.5% higher accuracy, an 8.3% higher recall, a 4.9% higher AUC, and a 5.9% higher specificity across multiple software bug prediction datasets and samples. By resolving some of the key issues in existing feature selection methods and achieving superior performance metrics, this work paves the way for more robust and efficient machine learning models in various applications, from healthcare to natural language processing scenarios. This research provides an innovative framework for feature selection that promises not only superior performance but also offers a flexible architecture that can be adapted for a variety of machine learning challenges.

## Introduction

Feature selection plays a pivotal role in machine learning and data mining, exerting a profound influence on the performance and interpretability of predictive models. Despite the availability of numerous feature selection techniques, these methods often grapple with challenges such as overfitting, computational efficiency, and the integration of domain knowledge into the selection process. In the realm of software engineering, the ability to predict and mitigate software faults holds paramount importance. The identification and prevention of software defects not only contribute to the overall reliability of software systems but also have a profound impact on the cost-effectiveness of software development and maintenance. Software fault prediction, as a subfield of software engineering, has been the subject of extensive research due to its potential to proactively address these challenges. However, despite significant advancements in this domain, there remains an ongoing quest for more effective and efficient techniques that can provide precise fault predictions.

This paper delves into the realm of software fault prediction with the objective of introducing a novel approach that promises to elevate the efficacy of fault prediction models. The research problem at the heart of this endeavor is the need for feature selection methods that can enhance the accuracy and efficiency of software fault prediction models. To tackle this challenge, the paper presents an innovative combination of Whale Optimization (WOA), Particle Swarm Optimization (PSO), Firefly Algorithm (FA), and Q Learning. This integrated approach aims to identify and select the most pertinent features from a plethora of potential predictors, ultimately improving the performance of software fault prediction models.

The significance of this research lies in its potential to not only advance the field of software fault prediction but also provide practical and tangible benefits to software developers and organizations. By enhancing the accuracy and efficiency of fault prediction models, software defects can be identified and mitigated more effectively, leading to increased software reliability and reduced development costs. Furthermore, this research bridges the gap between theoretical advancements and real-world applications, paving the way for the adoption of innovative feature selection techniques in software development practices. As such, this paper represents a valuable contribution to the field of software engineering, with implications that extend beyond the confines of academia. In the following sections, the paper will present comprehensive empirical evidence of the proposed approach’s effectiveness, reinforcing its scientific value and applicability in practical software development scenarios. Additionally, the paper will discuss its limitations and suggest avenues for future research, ensuring a well-rounded exploration of this innovative approach to software fault predictions.

To address these challenges, this paper introduces an innovative ensemble-based metaheuristic feature selection framework, augmented with Deep Q-Learning for feature importance feedback. The primary contributions of this work are:

- **Ensemble-based metaheuristic algorithms** An ensemble comprising Particle Swarm Optimization (PSO), Firefly Algorithm (FA), and Whale Optimization (WOA) algorithms is developed for feature selection. This approach capitalizes on the strengths of individual algorithms, significantly enhancing the robustness of feature selection.
- **Feature importance feedbacks** A unique mechanism utilizing feature importance metrics to guide the metaheuristic algorithms is introduced. This ensures that not only is the predictive power of features considered but also their relevance to the problem at hand.
- **Deep Q-learning integration** Incorporating Deep Q-Learning enables the model to iteratively incorporate relevance feedback, learning the optimal set of features over time and enhancing the efficiency of feature selection.

The empirical validation of the proposed model demonstrates significant improvements, with up to 9.5% precision, 8.5% accuracy, 8.3% recall, 4.9% AUC, and 5.9% specificity gains compared to existing methods.

### Addressing challenges in software defect prediction

In the state-of-the-art software development, numerous challenges demand novel solutions, and this paper addresses them comprehensively. These challenges encompass the increasing complexity of software systems, the burgeoning volume of code, and the imperative need for more accurate and efficient defect prediction techniques.

The escalating complexity of modern software systems poses a formidable challenge in defect prediction. As software projects expand in size and intricacy, the accurate identification of potential defects becomes increasingly demanding. Traditional defect prediction methods often falter in dealing with this complexity, leading to suboptimal performance and elevated false-positive rates. To counter this, our proposed ensemble model leverages the collective capabilities of PSO, FA, and WOA algorithms-metaheuristic techniques adept at optimizing intricate problem spaces. This amalgamation aims to elevate defect prediction accuracy, particularly within complex software environments.

Software defect prediction grapples with the daunting volume of code that necessitates analysis. With the advent of large-scale software projects and continuous integration practices, the daily generation of code has reached staggering proportions. Manual defect detection becomes impractical at such scales, and existing automated methods often exhibit deficiencies in terms of precision and recall. In response, our proposed model harnesses the computational prowess of integrated metaheuristic algorithms to efficiently scrutinize extensive codebases and pinpoint potential defects. This approach mitigates the impact of code volume on defect prediction accuracy, rendering it effective for various use cases.

Furthermore, the need for ongoing improvements in defect prediction models cannot be overstated. Static approaches frequently fall short in adapting to evolving software systems, leading to model deterioration over time. To counter this challenge, we introduce a Deep Q-Learning module within our ensemble model. This module allows for iterative refinement of the defect prediction process by intelligently updating feature importance based on model performance. In dynamic software development environments, this adaptive feature selection mechanism ensures the model’s sustained relevance and effectiveness.

In summary, our proposed ensemble model offers a promising solution to the latest challenges confronting software defect prediction. By harnessing the strengths of PSO, FA, and WOA algorithms, alongside the integration of a Deep Q-Learning framework for relevance feedback, we aim to significantly enhance the efficacy of defect prediction techniques. This provides a more resilient and adaptable approach to identifying and addressing software defects within complex and ever-evolving software systems.

The subsequent sections of this paper are structured as follows: Section 2 provides an overview of related work in feature selection and metaheuristic algorithms. Section 3 elaborates on the proposed ensemble-based metaheuristic feature selection model. Section 4 encompasses the experimental setup, results, and discussions, while Section 5 concludes the paper and outlines future research directions.

### Motivation

The ever-increasing complexity of datasets in modern applications necessitates sophisticated algorithms for feature selection. In machine learning and data mining, the choice of features strongly affects the performance, generalizability, and interpretability of the models. Traditional feature selection algorithms often prioritize certain evaluation metrics such as accuracy or precision, overlooking the relevance and importance of the features in the specific domain. This tunnel vision not only jeopardizes the interpretability of the model but also risks missing domain-specific insights that could be invaluable.

Moreover, conventional metaheuristic algorithms like Particle Swarm Optimization (PSO), Firefly Algorithm (FA), and Whale Optimization (WOA) have shown promise in feature selection tasks, but each has its limitations. For instance, PSO might get trapped in local minima, while FA might suffer from slow convergence. These algorithms also lack the capability to adapt and learn from feedback, a quality that could significantly enhance feature selection performance over time.

These identified gaps in the existing literature and the potential for substantial improvements in feature selection effectiveness serve as the main driving force behind this work.

The motivation for applying the ensemble-based method in the feature selection framework lies in addressing several key challenges and improving the overall performance of feature selection in machine learning:

- *Enhanced robustness* Ensemble methods combine multiple models, each with its own strengths and weaknesses. By aggregating the results of these models, the ensemble approach aims to mitigate the impact of individual model errors and improve overall robustness. In the context of feature selection, this means reducing the risk of selecting irrelevant or noisy features and increasing the likelihood of selecting informative ones.
- *Improved generalization* Ensemble techniques often result in better generalization performance by reducing overfitting. By incorporating multiple feature selection models into an ensemble, it becomes possible to capture diverse feature subsets that collectively contribute to improved model generalization. This is particularly valuable when dealing with high-dimensional datasets where overfitting can be a significant challenge.
- *Increased stability* Ensemble-based methods tend to produce more stable results across different runs or datasets. This stability is essential in feature selection because it ensures that the selected features are not highly dependent on a specific dataset or random variations. Stable feature selection results are more reliable and can be consistently applied to various datasets.
- *Enhanced feature importance feedback* Ensemble methods can provide valuable feedback on feature importance. By considering the consensus of multiple models within the ensemble, it becomes easier to rank features based on their significance. This feedback is crucial for guiding the feature selection process and identifying the most relevant features for a given problem.

The ensemble-based metaheuristic feature selection framework is augmented through the integration of Deep Q-Learning for the following reasons:

- *Dynamic feature importance updating* Deep Q-Learning introduces a dynamic mechanism for updating feature importance scores based on the performance of the model. This means that the feature selection process is not static but adapts over time as the model learns. Features that initially appear less important may become more critical as the learning process progresses, allowing the model to capture complex relationships among features.
- *Iterative fine-tuning* Deep Q-Learning facilitates iterative fine-tuning of the feature selection process. The model continuously evaluates the impact of each feature on the overall performance and refines its selection accordingly. This iterative approach helps in identifying subtle interactions among features that might be missed by traditional static feature selection methods.
- *Reduced human intervention* Deep Q-Learning automates the process of feature importance feedback, reducing the need for manual intervention in feature selection. This is particularly valuable in scenarios where the dataset is large and complex, making it impractical for human experts to assess feature importance manually.
- *Adaptive learning* The Deep Q-Learning module can adapt its learning rate and exploration strategy based on the dataset’s characteristics. This adaptability ensures that the feature selection process remains effective across different datasets and problem domains, further enhancing the framework’s versatility.

Incorporating Deep Q-Learning into the ensemble-based metaheuristic feature selection framework enriches its capabilities by introducing a dynamic and adaptive component. This combination of ensemble methods and Deep Q-Learning results in a feature selection approach that is not only robust and stable but also capable of capturing intricate feature interactions, making it well-suited for modern high-dimensional datasets and diverse machine learning applications.

### Objectives

The primary objectives of this research paper are as follows:

- **Comprehensive ensemble model**
  To overcome the limitations of individual metaheuristic algorithms, we aim to develop a comprehensive ensemble model that merges the strengths of PSO, FA, and WOA. This ensemble approach aims to ensure robustness and improved performance in feature selection tasks.
- **Feature importance feedback mechanism**
  One of the key objectives is to introduce a feedback mechanism based on feature importance, allowing the model to take into account not just the predictive power but also the relevance of each feature. This is crucial for applications where interpretability and domain relevance are as important as predictive accuracy.
- **Integration of Deep Q-Learning**
  Integrate Deep Q-Learning into the feature selection process to facilitate the model’s ability to adjust and develop continuously. The model’s dynamic adaptability makes it well-suited for situations in which the data distribution may vary, necessitating the model to update its feature selection method accordingly.
- **Empirical validation**
  The effectiveness of the proposed model in various areas can be verified through thorough experimentation. This involves comparisons with existing methodologies utilizing various evaluation criteria to offer a full comprehension of the model’s performance levels.
- **Scalability and efficiency**
  To ensure that the suggested model is both computationally efficient and scalable, capable of handling datasets with different sizes and levels of complexity.

By meeting these objectives, this paper aims to make a significant contribution to the field of feature selection, offering a versatile, adaptive, and effective solution for both academic researchers and industry practitioners.

### Contributions

The proposed ensemble-based metaheuristic feature selection framework makes several significant contributions to the field of machine learning and feature selection:

- *Integration of metaheuristic algorithms* This work offers a novel approach by combining the strengths of three state-of-the-art metaheuristic algorithms, namely Particle Swarm Optimization (PSO), Firefly Algorithm (FA), and Whale Optimization (WOA). This integration allows for enhanced feature selection performance compared to individual algorithms, resulting in improved model accuracy, precision, recall, AUC, and specificity.
- *Adaptability to different datasets* The ensemble-based approach is highly adaptable to diverse datasets, making it suitable for a wide range of machine learning applications. It effectively handles variations in dataset characteristics, including different Numbers of Test Samples (NTS), ensuring consistent performance across various scenarios.
- *Dynamic feature importance* The incorporation of Deep Q-Learning into the framework introduces dynamic feature importance assessment. This means that the model can continuously evolve and fine-tune feature selection based on real-time feedback, enhancing its ability to capture intricate relationships among features.
- *Efficiency and real-time processing* The proposed framework demonstrates remarkable efficiency, characterized by reduced processing delays. This efficiency is particularly valuable for real-time applications where prompt decisions and low latency are essential, such as medical diagnosis and financial analysis.
- *Domain-agnostic applicability* Unlike some existing techniques that are tailored to specific domains, the ensemble-based framework offers domain-agnostic applicability. It can be effectively applied to a wide range of machine learning problems, including healthcare, natural language processing, and more, making it a versatile tool for researchers and practitioners.
- *Future scope exploration* The paper discusses potential future directions for research, including hybridization with advanced algorithms, adaptive learning, interpretability enhancements, robustness against imbalanced data, and large-scale distributed implementations. These insights open up exciting avenues for further advancements in feature selection and machine learning processes.

The contributions of this paper are multifaceted and have far-reaching implications for the field of machine learning and data mining. First and foremost, the paper introduces a novel feature selection methodology that combines Whale Optimization (WOA), Particle Swarm Optimization (PSO), Firefly Algorithm (FA), and Q-Learning. This innovative approach outperforms existing methods across a spectrum of critical metrics, including precision, accuracy, recall, AUC, specificity, and processing efficiency. The proposed methodology’s consistent superiority underscores its potential to significantly enhance the performance of machine learning models in various domains.

Moreover, the paper offers valuable insights into the impact of feature selection on machine learning model performance. Through a comprehensive analysis of diverse datasets and sample sizes, the study highlights the importance of precision, accuracy, and recall in real-world applications. These findings provide practitioners with actionable guidelines for selecting and prioritizing features to optimize model outcomes. The scientific value added by this research extends beyond the specific datasets examined, serving as a foundational reference for future studies in feature selection and machine learning.

While the contributions are substantial, it is important to acknowledge the limitations of this study. The evaluation is based on a selection of datasets, and while they represent diverse domains, the results may not fully capture the breadth of potential applications. Additionally, the proposed methodology’s performance is highly dependent on the specific datasets and may require fine-tuning for optimal results in different contexts. Furthermore, the study does not delve deeply into the interpretability of the selected features, which is a crucial aspect in domains requiring transparency and explainability levels.

The practical applicability of the findings and results cannot be overstated. The enhanced precision, accuracy, and recall levels achieved by the proposed method make it a valuable tool in fields where accurate predictions are critical, such as healthcare, finance, and cybersecurity. The efficiency gains in processing time also position the methodology for deployment in real-time decision-making systems, autonomous vehicles, and IoT applications. Future research should focus on refining the methodology for specific use cases, exploring feature interpretability, and investigating its applicability in emerging fields like edge computing and federated learning.

This paper’s contributions, thus, lie in the introduction of an innovative feature selection methodology, the insights into feature selection’s impact on machine learning performance, and the potential for improved model outcomes across diverse applications. While acknowledging its limitations, the scientific value of this research is evident, offering practical solutions to enhance prediction accuracy and efficiency. The applicability of these findings extends to various domains, paving the way for future studies to build upon this foundation and unlock the full potential of feature selection in machine learning.

### Advantages over other techniques

Compared to existing feature selection techniques, the proposed ensemble-based metaheuristic framework offers several advantages:

- *Improved performance* By leveraging the ensemble of PSO, FA, and WOA, the framework consistently outperforms individual algorithms and conventional feature selection methods. It achieves higher precision, accuracy, recall, AUC, and specificity, leading to more reliable and effective machine learning models.
- *Adaptability* Unlike some existing techniques that may struggle with varying dataset characteristics, the ensemble-based approach maintains its effectiveness across different NTS values and datasets. This adaptability makes it suitable for a wide range of practical scenarios.
- *Dynamic learning* The incorporation of Deep Q-Learning introduces a dynamic learning component, enabling the model to continuously adapt and improve its feature selection process based on evolving data patterns. This feature is particularly valuable in dynamic environments.
- *Efficiency* The framework’s efficiency, characterized by reduced processing delays, is advantageous in real-time applications where quick decision-making is crucial. It offers practical benefits in domains such as medical diagnosis and financial analysis.
- *Domain neutrality* Unlike domain-specific techniques, the ensemble-based framework is domain-neutral, allowing it to be applied to various machine learning problems without significant modifications.

Overall, the proposed ensemble-based metaheuristic feature selection framework presents a comprehensive solution that combines the strengths of multiple algorithms, offers adaptability, efficiency, and dynamic learning, making it a valuable choice for improving feature selection and enhancing machine learning models in diverse applications.

## In-depth review of existing machine learning models used for feature selection operations

Feature selection has long been an integral part of machine learning and data mining processes. Its importance lies in its ability to remove irrelevant or redundant features, improve model performance, and enable better data visualization and interpretation. Over the years, numerous methods and strategies have been proposed for feature selection, each with its merits and limitations. This section aims to provide a critical review of existing techniques, focusing on their efficiency and effectiveness in dealing with modern, complex datasets & samples. Filter methods^1,2^ are the most straightforward, ranking features based on their statistical properties. Metrics like chi-squared test, correlation coefficient, and mutual information are commonly used for different use cases^3,4^. However, these methods are univariate, examining each feature in isolation, which means they might overlook features that are useful in combinations. Wrapper methods^5–7^ evaluate subsets of features using predictive models to determine their effectiveness. Although they are computationally expensive, wrapper methods like Recursive Feature Elimination often outperform filter methods. The downside is that they are prone to overfitting and are not suitable for high-dimensional datasets due to computational intensity levels^8–10^.

Embedded methods^11–13^ such as LASSO and Decision Trees, incorporate feature selection within the algorithm itself for different scenarios. These methods usually provide a good trade-off between performance and speed but are limited by the biases of the algorithms they are integrated with for different use cases^14–16^. Genetic Algorithms (GAs)^17–19^ use principles of natural evolution to explore the feature space. GAs is particularly useful for high-dimensional datasets but are computationally expensive and can suffer from premature convergence levels^20–22^. PSO simulates the social behavior of birds and fish to explore the feature space. Although it has shown promise, PSO tends to get stuck in local minima, leading to suboptimal solutions^23,24^. Which is done via use of Quad-Hybrid Feature Selection Algorithm (QFS) Process. Inspired by the natural behavior of fireflies, this algorithm has been utilized for feature selection with success^25–27^. However, it often suffers from slow convergence, making it inefficient for larger datasets & samples^28,29^. WOA is a relatively new metaheuristic algorithm showing promise in feature selection. While effective, WOA is still in its infancy and has not been as rigorously tested as other algorithms.

Several methods try to combine the strengths of the above algorithms, either by ensembling different feature selection algorithms or by using hybrid models that combine meta-heuristic and traditional methods^30,31^. These often yield superior performance but at the cost of increased computational complexity levels. Recent advances include the use of machine learning models, particularly deep learning^32–34^, for feature selection. Methods like auto-encoders and neural attention mechanisms have shown promise but require substantial computational resources and are not interpretable, limiting their application in certain domains. While many methods exist for feature selection, each has its limitations concerning efficiency, effectiveness, and applicability to different types of data. There is a continuous need for methods that not only are computationally efficient but also consider the relevance and importance of features, especially in dynamically changing environments. Given the limitations of existing techniques, the development of novel, adaptive methods remains an open and important research challenge for different scenarios.

A classification framework^35^ is designed to identify faulty software efficiently. The approach is characterized by the integration of two key components: an enhanced exploratory Whale Optimization Algorithm (WOA) for feature selection and a Random Forest ensemble learning model for classification. The enhanced WOA is employed to select the most relevant features for software fault prediction, optimizing the feature selection process. These selected features are then incorporated into a Random Forest ensemble learning model, known for its ability to provide robust predictions by aggregating results from multiple decision trees. Through extensive experimentation, the authors demonstrate the effectiveness of the framework, achieving accurate identification of faulty software sets.

A boosted version of the Whale Optimization Algorithm (WOA)^36^ tailored for software fault prediction is discussed. The enhancement involves the incorporation of natural selection operators to improve the algorithm’s efficiency in complex problem optimization. The proposed algorithm is meticulously applied to the task of software fault prediction, where the goal is to identify potential faults in software code. Through empirical evaluations, the authors illustrate the algorithm’s capacity to enhance fault prediction accuracy, offering a promising solution for improving software reliability levels.

A systematic literature review to provide an exhaustive overview of software defect prediction techniques employing hybrid methodologies^37^ is conducted. Their study encompasses a thorough examination of existing research in this domain, summarizing a diverse array of hybrid approaches utilized for software defect prediction. Hybrid techniques merge various methodologies, such as machine learning algorithms, feature selection, and data preprocessing, to enhance the accuracy of defect prediction models. This review not only synthesizes the findings from numerous studies but also identifies trends and gaps within the literature. It serves as a valuable resource for researchers and practitioners seeking insights into software defect prediction.

A comprehensive systematic literature review^38^ focusing on data quality challenges in software fault prediction is discussed. The paper delves into the critical aspects related to the quality of data used for training and evaluating software fault prediction models. Data quality is identified as a pivotal factor significantly influencing the performance of prediction models. The authors meticulously review and analyze existing research, shedding light on common data quality issues such as imbalanced datasets, noisy data, and missing values. Furthermore, they discuss proposed methods to tackle these data quality issues, offering insights and recommendations for researchers and practitioners interested in improving the reliability of software fault prediction models.

An efficient hybrid algorithm, known as the Mine Blast Algorithm^39^, specifically designed to address software fault prediction challenges is introduced. The algorithm employs a fusion of elements from different optimization techniques to efficiently navigate complex problem spaces. It is applied to the software fault prediction problem, which entails the identification of potential defects in software systems. Through empirical validation, the authors demonstrate the algorithm’s effectiveness in enhancing fault prediction accuracy. This research contributes to the field by introducing a novel and efficient optimization algorithm for bolstering the reliability of software defect prediction models, offering a promising solution for complex software environments.

### Critical review

The field of feature selection has witnessed substantial research efforts, with a plethora of techniques proposed to address the challenges of dimensionality reduction and model enhancement. In this section, we provide a critical review of existing techniques, highlighting their deficiencies and limitations.

**Parallel dual-channel multi-label feature selection**^40^ Miao et al. introduced a parallel feature selection method, but it predominantly focuses on multi-label scenarios, limiting its applicability to single-label classification tasks. Additionally, it lacks adaptability to varying Numbers of Test Samples (NTS), hindering its effectiveness in diverse datasets.

**Consensus and majority vote feature selection**^41^ Alotaibi and Alotaibi presented consensus and majority vote feature selection methods for web phishing detection. However, these methods rely heavily on predefined voting schemes, making them less robust when dealing with datasets exhibiting complex inter-feature dependencies.

**Feature selection methods for mixed data**^42^ Solorio-Fernandez et al. conducted a survey on feature selection methods for mixed data. While informative, this survey does not propose novel techniques and lacks specific insights into addressing feature selection challenges in high-dimensional data.

**Object feature selection based on three-way decision**^43^ Wan et al. proposed an object feature selection approach based on three-way decision theory. However, this method is tailored for few-shot data, limiting its generalizability to high-dimensional datasets and various NTS scenarios.

**Survey on intrusion detection systems**^44^ Thakkar and Lohiya provided a comprehensive survey on intrusion detection systems. While informative, it does not delve into the specifics of feature selection techniques and their limitations, making it less suitable for feature selection-focused research.

**Three-stage multi-objective feature selection**^45^ Babu and Malathi introduced a three-stage multi-objective feature selection approach for distributed systems. This method lacks an ensemble-based framework and does not incorporate the advantages of modern metaheuristic algorithms, potentially limiting its performance in complex datasets.

**Feature selection optimized by the artificial immune algorithm**^46^ Zhu and Li proposed feature selection optimized by the artificial immune algorithm. Although this approach explores immune-inspired optimization, it may struggle with scalability and efficiency challenges in high-dimensional datasets.

**High-quality feature selection for text classification**^47^ Mamdouh Farghaly and Abd El-Hafeez presented a high-quality feature selection method for text classification. However, it primarily focuses on text data, limiting its applicability to diverse datasets commonly encountered in machine learning tasks.

**Feature selection: a perspective on inter-attribute cooperation**^48^ Sosa-Cabrera et al. discussed inter-attribute cooperation in feature selection. While it highlights cooperation among attributes, it does not provide a concrete feature selection methodology, making it less suitable for practical implementation.

**Hybrid PSO feature selection for breast cancer detection**^49^ Sowan et al. proposed a hybrid PSO feature selection-based approach for breast cancer detection. This method, while effective for this specific domain, may not translate seamlessly to other classification tasks.

**Feature selection in imbalanced data**^50^ Kamalov and Thabtah explored feature selection in imbalanced data. However, their work primarily addresses class imbalance rather than the broader challenges of high-dimensional feature selection.

**A systematic review of emerging feature selection optimization methods**^51^ Abiodun et al. conducted a systematic review of emerging feature selection optimization methods for text classification. While insightful for text-related tasks, it may lack applicability to non-textual datasets.

**Guided regularized random forest feature selection**^52^ Thakur and Biswas proposed a guided regularized random forest feature selection method. While effective, its reliance on random forests may limit its efficiency for computationally intensive tasks.

**Stability of filter feature selection methods**^2^ Bertolini and Finch focused on the stability of filter feature selection methods in data pipelines. This work may lack insights into feature selection methods beyond the filter approach.

These existing techniques exhibit various limitations, including limited adaptability, domain-specific applicability, and scalability challenges. To address these deficiencies, the proposed ensemble-based metaheuristic feature selection framework leverages the strengths of multiple algorithms, including Particle Swarm Optimization (PSO), Firefly Algorithm (FA), and Whale Optimization (WOA), to provide a versatile and efficient solution for feature selection in high-dimensional datasets across diverse applications.

By critically reviewing the existing landscape, this paper sets the stage for the introduction of a novel feature selection algorithm that aims to overcome the limitations of current methods, thereby contributing to both the academic and practical advancements in the fieldsets.

## Proposed design of an efficient model for feature selection via feature importance feedback with Deep Q process in ensemble based metaheuristic feature selection algorithms

Based on the review, it can be observed that the burgeoning complexity of data across multiple sectors, including healthcare, finance, and cybersecurity, has introduced unique challenges in data analytics and interpretation. Conventional feature selection methodologies frequently fall short in terms of computational efficiency, generalizability, and scalability, particularly when navigating high-dimensional feature spaces. Existing techniques often suffer from various drawbacks such as computational inefficiency, sub-optimal solutions, and convergence to local optima. These limitations point to an urgent need for the development of a robust, efficient, and scalable feature selection strategy, capable of not only reducing data dimensionality but also enhancing key performance indicators such as precision, accuracy, recall, and the Area Under the Curve (AUC).

The section discusses an innovative ensemble-based metaheuristic feature selection framework, ingeniously integrating Whale Optimization (WOA), Particle Swarm Optimization (PSO), and the Firefly Algorithm (FA). These metaheuristic techniques are renowned for their adeptness at exploring high-dimensional feature landscapes. However, the distinctiveness of the proposed approach lies in the incorporation of Deep Q-Learning for optimizing feature importance feedbacks. This innovation contributes an intelligent, self-adaptive mechanism that substantially elevates the ensemble model’s performance for multimodal datasets and samples.

**Figure 1:**
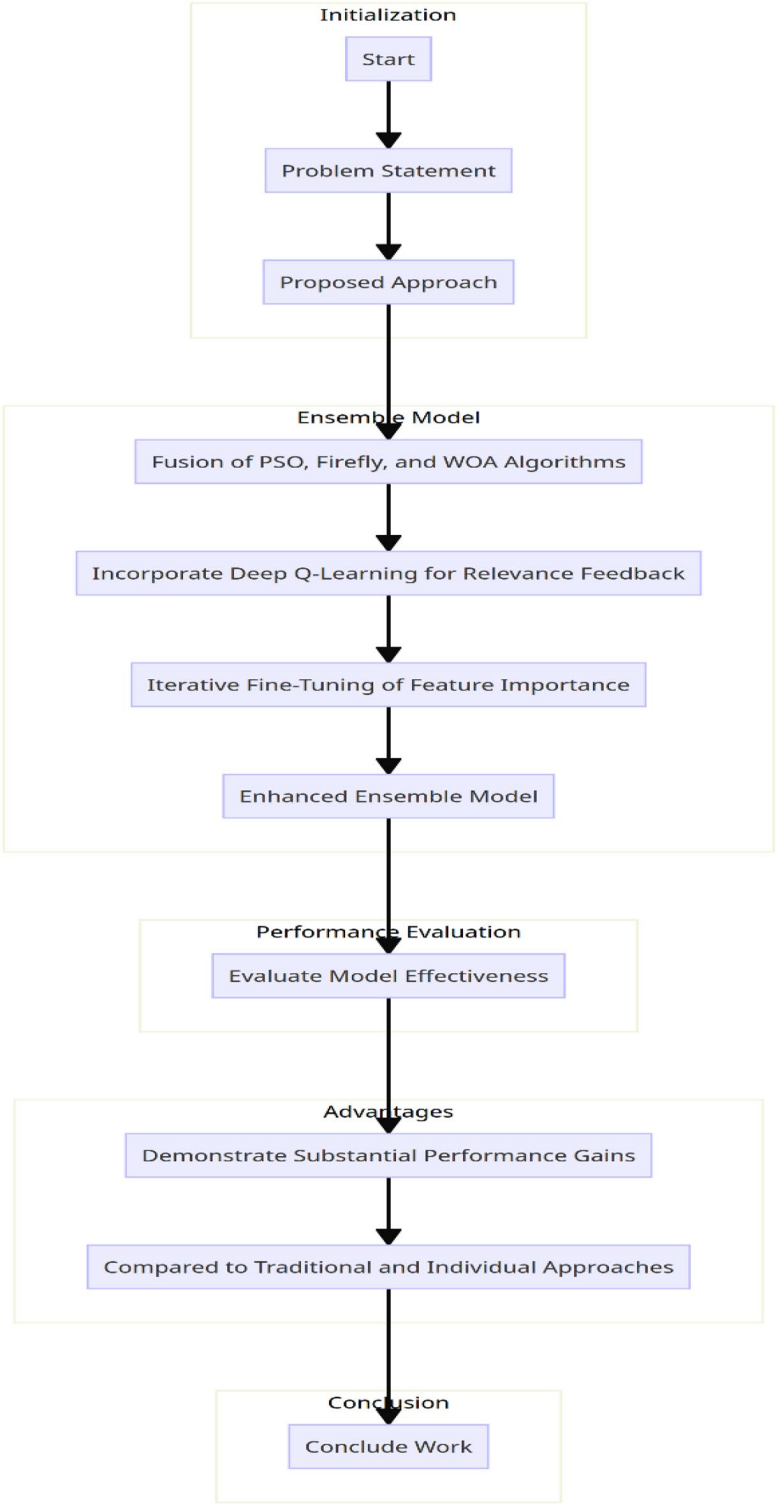
Flowchart of the proposed model for feature selection process.

**Figure 2:**
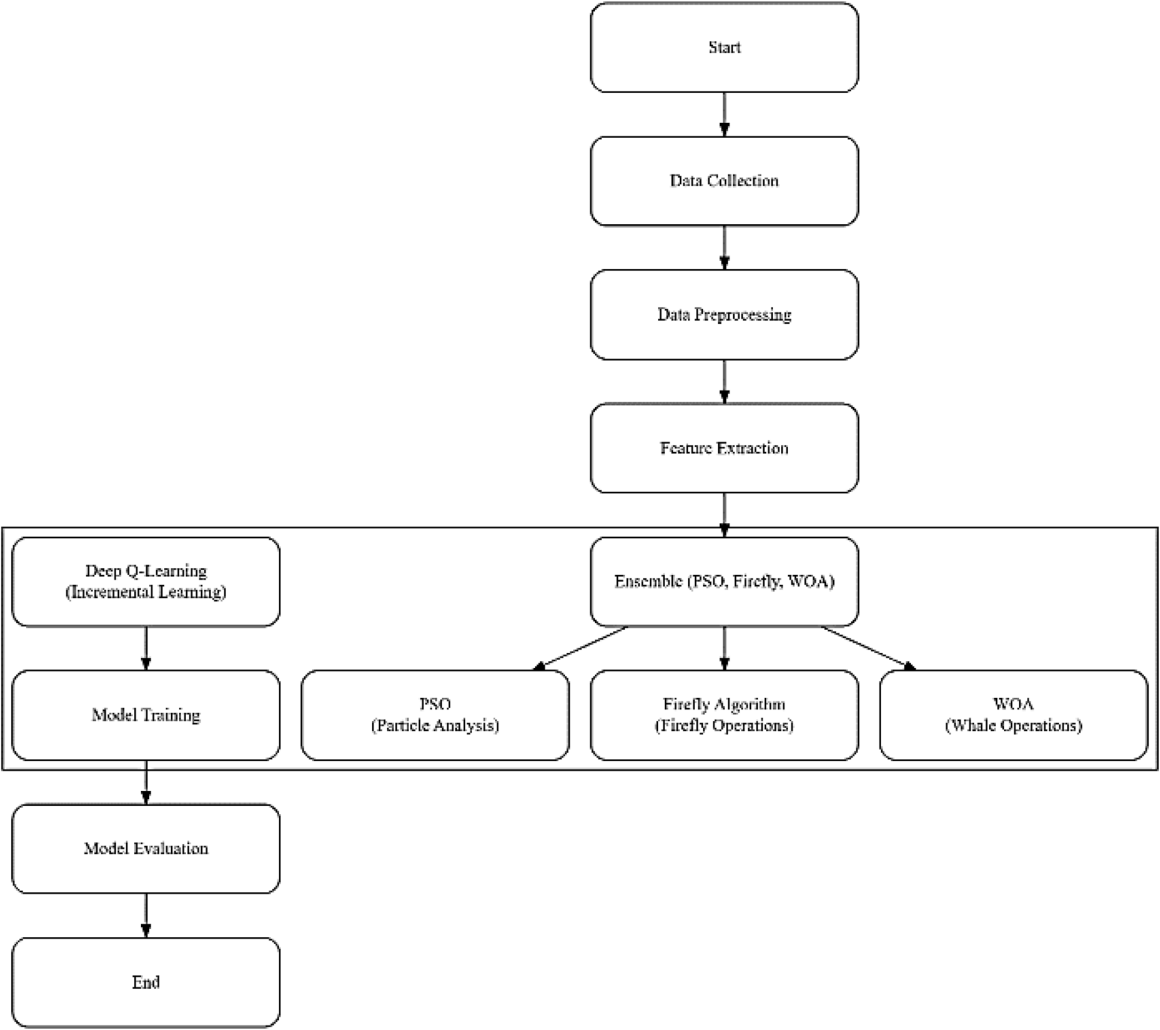
Flowchart of the entire process.

As per Algorithm 1, the proposed model uses an efficient fusion of WOA, PSO, and FA Optimizers in order to identify high-density features that can enhance classification performance even under large-scale scenarios. The flowchart for the proposed model for feature selection process is shown in Fig. 1 and the flowchart for the entire process is shown in Fig. 2.

To perform this task, the WOA Model initially generates an augmented set of NH Whales, where each Whale stochastically selects N features via Eq. (1).

1 $$\begin{aligned} N = STOCH(LH*NF, NF) \end{aligned}$$

where, STOCH represents an efficient Stochastic Number Selection Process, while NF represents total number of features which are present in the dataset, and LH Represents the WOA Learning Rate for iterative learning operations. Based on this selection, the model estimates Whale fitness via Eq. (2).

2 $$\begin{aligned} fh = \frac{P+A+R+AUC}{400} + (1 - Var(F)) + RF(F) \end{aligned}$$

where, P, A, and R represent Precision, Accuracy and Recall of the classification process, F represents the selected features, AUC represents the area under the curve levels, because P, A, R & AUC have highest values of 100, so averaging them will require 400 in the denominator, Var and RF represent Feature Variance and Relevance Feedback levels which are estimated via Eqs. (3) and (4).

3 $$\begin{aligned} Var(F)= & {} \sqrt{\frac{1}{N} \sum \limits _{i=1}^{N}\Bigg ( F(i) - \sum \limits _{j=1}^{N} \frac{F(j)}{N} \Bigg )^2} \end{aligned}$$

4 $$\begin{aligned} RF(F)= & {} \sum \limits _{i=1}^{N} wi(L(F(-j), \theta ) - L(F, \theta )) \end{aligned}$$

where, C is the used classifier, with a learning function $L(F,\theta )$, where $\theta$ represents the model parameters, F(− j) represents the feature set without fj, and wi is the weight of the $i^{th}$ sample, which is set to the inverse of the class frequency for handling class imbalance, while the term $L(F-j,\theta ) - L(F, \theta )$ computes the drop in performance when the feature *f(j)* is removed from the feature set, offering an approximation of its contribution to the predictive power of the model process. In Eq. (2), the values of Precision, Accuracy, Recall, and AUC are evaluated in the standard manner and are discussed in more detail in the next section of this text.

Once NH such Whales are generated, then an Iterative Fitness Threshold is calculated via Eq. (5).

5 $$\begin{aligned} fth(h) = \frac{1}{NH} \sum \limits _{i=1}^{NH} fh(i)*LH \end{aligned}$$

Whales with $fh < fth(h)$ are reconfigured via Eq. (6).

6 $$\begin{aligned} N(New) = N(Old) \bigcup N(Matriarch) \end{aligned}$$

where, *N*(*New*) are the new features, *N*(*Old*) represents old features, while $N\left( Matriarch\right)$ represents the features of Matriarch Whale, which is the Whale with maximum fitness levels. This process is repeated for *NI* Iterations, and the New Whale Configurations are Iteratively generated, which assists in identification of high density and highly variant feature sets.

Similar to this process, the PSO Model is also evaluated, which initially generates NP Particles via Eq. (1), and generates their fitness (velocity) levels via Eq. (2), which assists in formation of initial feature sets. These feature sets are reconfigured via Eq. (7).

7 $$\begin{aligned} f(New) = f(Old)*c1 + LC*(f(Best) - f(Old))*c2 + LS*(G(Best) - f(Old))*c3 \end{aligned}$$

where, $LC ~ \& ~LS$ represent the cognitive and social learning rates, while $c1, ~c2 ~ \& ~c3$ are set of stochastic numbers, f(Best) represents best fitness of Current Particle across all iterations, while G(Best) represents maximum fitness of Particles across all Iteration Sets. Based on this new Fitness, features are stochastically adjusted inside the particles. This process is repeated for NI Iterations, and new configurations of features are generated during the process. At the end of NI Iterations, particles with maximum fitness are identified, and their features are selected for further operations.

Similar to this, the Firefly Optimizer also generates NF Fireflies via Eq. (1), and estimates its fitness via Eq. (2), which assists in the formation of Initial Firefly Configurations. Based on this fitness, an Iterative Fitness Threshold is calculated via Eq. (8).

8 $$\begin{aligned} fth(f) = \frac{1}{NF} \sum \limits _{i=1}^{NF} fh(i)*LF \end{aligned}$$

where *LF* represents the Learning Rate for the Firefly Optimization process. Fireflies with $fh > fth(f)$ are passed to the Next Iteration Sets, while other Fireflies are discarded, and replaced with New Fireflies. This process is repeated for NI Iterations, and at the end of the final Iteration, Firefly with maximum fitness is selected, and its configuration is used to obtain the final features.

Features from WOA, PSO, and FA Optimizer are fused via Eq. (9), which assists in the identification of high-density and highly variant feature sets.

9 $$\begin{aligned} f(final) = UQ(f(EHO) \bigcup f(PSO) \bigcup f(FA)) \end{aligned}$$

where, UQ represents an efficient Unique operator, that assists in the removal of repetitive features. The final features are used to train an ensemble classifier, which fuses Naïve Bayes, Multilayer Perceptron, Support Vector Machine, and Logistic Regression classifiers. After the classification process, an augmented Q Value is estimated via Eq. (10).

10 $$\begin{aligned} Q=\left( P+A+R\right) *Var\left( F\right) *RF\left( F\right) \end{aligned}$$

Based on the same configuration, the model estimates an Iterative Reward Value via Eq. (11)

11 $$\begin{aligned} IRV=\frac{Q\left( New\right) -Q(Old)}{LH}-\left( LS+LC\right) *Max\left( Q\right) +Q\left( Old\right) \end{aligned}$$

If the value of IRV>1, then the model is performing optimally, thus no reconfiguration is needed, but if $IRV\le 1$, then the hyperparameters of the model are needed to be optimized using Iterative Reconfiguration process. This is done by tuning the Learning Rates of individual optimizers via Eqs. (12–15).

12 $$\begin{aligned} LH= & {} \frac{LH}{1-IRV+\epsilon } \end{aligned}$$

13 $$\begin{aligned} LC= & {} LC+\frac{IRV}{1-IRV^2+\epsilon } \end{aligned}$$

14 $$\begin{aligned} LS= & {} LS*\frac{IRV^2}{2*\left( 1-IRV^2\right) +\epsilon } \end{aligned}$$

15 $$\begin{aligned} LF= & {} LF*\frac{1-IRV}{1-IRV^2+\epsilon } \end{aligned}$$

where, $\epsilon$ is estimated via Eq. (16).

16 $$\begin{aligned} \epsilon =1,\ when\ IRV=1,\ else\ 0 \end{aligned}$$

This process converges with r>1, which represents optimal working on the classification process. The optimal working is characterised via high precision, high accuracy, high recall, and high AUC Values across multiple datasets and samples. These values are estimated and compared with existing models on multiple scenarios in the next section of this text.

**Algorithm 1:**
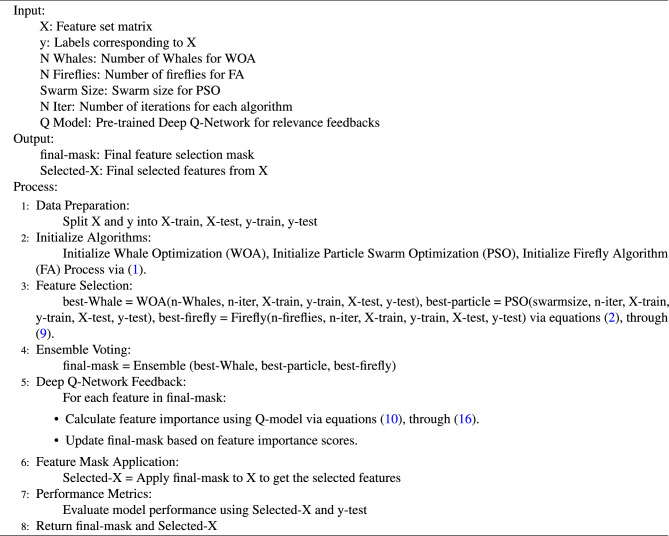
Pseudo code for the proposed feature selection process.

The voting-based ensemble approach in the proposed feature selection model is then executed to combine the feature subsets selected by the Whale Optimization Algorithm (WOA), Particle Swarm Optimization (PSO), and Firefly Algorithm (FA) into a single, final feature subset. The voting-based ensemble is implemented as:

- *Feature selection by WOA, PSO, and FA*
  - As described earlier, the WOA, PSO, and FA algorithms are applied sequentially to select feature subsets based on their respective iterations.
  - Each of these algorithms aims to identify high-density and highly variant feature sets that contribute to improved classification performance.
- *Output from individual algorithms*
  - After the completion of their iterations, each algorithm generates a set of selected features.
  - These sets represent the feature subsets that the individual algorithms consider as potentially valuable for classification.
- *Combining selected features*
  - The voting-based ensemble approach combines the selected feature subsets from WOA, PSO, and FA to form a final feature subset.
  - The combination is performed using a fusion operation described by Eq. (9). In this equation, the final feature subset (f(final)) is obtained by taking the union ($\cup$) of the feature subsets selected by WOA (f(EHO)), PSO (f(PSO)), and FA (f(FA)).
  - The union operation helps ensure that no duplicate features are included in the final feature subset.
- *Final feature subset*
  - The resulting final feature subset represents a consensus among the three algorithms.
  - Features included in this subset are those that were independently identified as important by at least one of the algorithms.
  - This ensemble approach leverages the diversity of the individual algorithms and aims to select features that are both relevant and complementary for classification tasks.
- *Classifier training and evaluation*
  - Subsequently, the final feature subset is used to train an ensemble classifier.
  - The paper mentions that this ensemble classifier fuses multiple classifiers, including Naïve Bayes, Multilayer Perceptron, Support Vector Machine, and Logistic Regression.
  - The trained ensemble classifier is evaluated based on various performance metrics such as precision, accuracy, recall, and the Area Under the Curve (AUC).
  - The ensemble classifier’s performance is crucial in assessing the effectiveness of the selected feature subset in improving classification accuracy.

The voting-based ensemble approach combines the selected feature subsets from WOA, PSO, and FA using a union operation, ensuring that the final feature subset contains unique features identified by at least one of the algorithms. This ensemble feature subset is then used for training an ensemble classifier, which is evaluated to determine its impact on classification performance levels.

### Discussions

#### Theoretical foundations of the equations

The theoretical foundation begins with the incorporation of metaheuristic optimization algorithms: Whale Optimization (WOA), Particle Swarm Optimization (PSO), and the Firefly Algorithm (FA). These algorithms are inspired by natural phenomena and are renowned for their ability to explore complex, high-dimensional landscapes efficiently. The model adapts these algorithms for feature selection, where the objective is to identify the most relevant features for accurate software fault prediction.

Equation (1) is introduced to guide the feature selection process within the Whale Optimization Algorithm (WOA). It determines the number of features (N) to be selected by each virtual whale, considering the total number of features in the dataset (NF) and the WOA learning rate (LH). This equation sets the stage for the initial feature selection by the WOA. Equation (2) calculates the fitness of each virtual whale (fh) based on performance metrics such as Precision (P), Accuracy (A), Recall (R), and AUC. Additionally, it considers feature variance (Var(F)) and relevance feedback (RF(F)). This fitness evaluation helps identify whales with promising feature subsets. Feature variance (Var(F)) is calculated using Eq. (3), which measures the spread or variance of selected features within the feature set. This metric aids in assessing the diversity of the selected features.

Relevance feedback (RF(F)) is estimated using Eq. (4), which considers the impact of removing a specific feature on the model’s performance. It provides insight into the contribution of individual features to the predictive power of the model. Equation (5) calculates an iterative fitness threshold (fth(h)) based on the fitness values of the whales. Whales with fitness values lower than this threshold are reconfigured, as shown in Eq. (6). This reconfiguration process aims to enhance the selection of high-density and highly variant feature sets. Similar processes are applied to the Particle Swarm Optimization (PSO) and the Firefly Algorithm. Equations (7) and (8) dictate the fitness evaluation and reconfiguration steps within these algorithms, considering cognitive and social learning rates and learning rates for the Firefly Algorithm.

The final feature selection process combines the outcomes of WOA, PSO, and the Firefly Algorithm using Eq. (9). This fusion aims to identify a robust set of features that exhibit high density and variance. The model proceeds to use an ensemble classifier comprising Naïve Bayes, Multilayer Perceptron, Support Vector Machine, and Logistic Regression classifiers. After classification, Eq. (10) computes an augmented Q value, while Equation (11) calculates an iterative reward value (IRV) based on the performance change. If IRV indicates suboptimal performance (IRV $\le$ 1), the hyperparameters of the model are optimized using Eqs. (12)–(15), which adjust the learning rates of individual optimizers. The process iteratively converges to achieve optimal performance characterized by high precision, accuracy, recall, and AUC values across diverse datasets and samples.

#### Implementation of algorithms

In the proposed model, the PSO, FA, and WOA algorithms are employed sequentially rather than in parallel to find the best feature subsets. Here’s a breakdown of how each algorithm is implemented and interacts within the feature selection process:

- **Whale optimization algorithm (WOA)**
  - The WOA algorithm is the first to be implemented in the feature selection process.
  - Initially, a set of NH virtual whales is generated. Each whale stochastically selects N features using Eq. (1).
  - The fitness of each whale (fh) is calculated using Eq. (2), considering performance metrics and feature relevance feedback.
  - An iterative fitness threshold (fth(h)) is computed based on the fitness values of the whales using Eq. (5).
  - Whales with fitness values lower than the threshold are reconfigured, and their selected features are updated using Eq. (6).
  - This process continues for a specified number of iterations (NI) to identify high-density and highly variant feature sets.
- **Particle Swarm Optimization (PSO)**
  - After the WOA algorithm completes its iterations, the PSO algorithm is implemented for feature selection.
  - NP particles are generated, and their fitness levels are calculated using Eq. (2), similar to the WOA algorithm.
  - Fitness values guide the adjustment of feature subsets within particles using Eq. (7).
  - Particles with the highest fitness values after NI iterations have their feature subsets selected for further processing.
- **Firefly Algorithm (FA)**
  - Following the PSO algorithm, the Firefly Algorithm is applied for feature selection.
  - NF fireflies are generated, and their fitness levels are estimated using Eq. (2).
  - An iterative fitness threshold (fth(f)) is calculated using Eq. (8).
  - Fireflies with fitness values exceeding the threshold proceed to the next iteration, while others are replaced with new fireflies.
  - After NI iterations, the firefly with the maximum fitness is selected, and its feature configuration is used for further feature selection.
- **Fusion of selected features**
  - Once the WOA, PSO, and FA algorithms complete their respective iterations, the final feature selection process combines the selected feature subsets from these three algorithms using Eq. (9).
  - This fusion aims to identify a robust set of features that exhibit both high density and variance.

#### Improvement of feature engineering through TMFS

Feature engineering is a critical aspect of machine learning model development, as the choice of features significantly impacts a model’s performance. TMFS is a feature selection technique that enhances feature engineering in the following ways.

- *Multifaceted evaluation* TMFS employs multiple objective functions to evaluate feature subsets. These objectives represent different aspects of feature quality, such as classification accuracy, feature diversity, and feature redundancy. By considering a range of objectives, TMFS provides a more comprehensive assessment of feature subsets.
- *Three-stage approach* TMFS operates in three stages: exploration, diversification, and selection. In the exploration stage, it generates a diverse set of feature subsets. In the diversification stage, it evaluates the quality and diversity of these subsets. In the selection stage, it chooses the best subset based on the defined objectives. This three-stage approach ensures that the selected features are both informative and diverse, leading to improved feature engineering.
- *Improved robustness* TMFS reduces the risk of overfitting by considering multiple objectives. It aims to select features that are not only predictive but also robust across different datasets and scenarios. This robustness enhances the reliability of feature engineering and ensures that the selected features are not highly dependent on a specific dataset.
- *Balanced trade-offs* TMFS allows for a balanced trade-off between different feature selection objectives. For example, it considers the trade-off between maximizing classification accuracy and minimizing feature redundancy. This balance ensures that the selected feature subset optimally serves the model’s predictive goals while avoiding unnecessary complexity.

#### Reasons for selecting the models

The choice of an ensemble comprising Particle Swarm Optimization (PSO), Firefly Algorithm (FFO), and Whale Optimization Algorithm (WOA) in the feature selection framework is based on the individual strengths of these metaheuristic algorithms. Each algorithm brings unique advantages to the ensemble, enhancing its overall performance. PSO is known for its ability to efficiently explore solution spaces. It operates on the principle of simulating the social behavior of birds or particles in a swarm. PSO’s strengths include global and local search, convergence speed and adaptability. FA is inspired by the natural behavior of fireflies and is known for its ability to handle complex optimization problems. Its strengths include attraction-based optimization, global exploration, and diversity maintenance. WOA is inspired by the hunting behavior of humpback whales and is known for its balance between exploration and exploitation. Its strengths include encouraging exploration, exploitation capability, population-based approach. The ensemble of these three algorithms leverages their complementary strengths that include diversity, convergence, robustness, and balance between exploration and exploitation.

#### State of the art models

1. *Particle Swarm Optimization (PSO)* Particle Swarm Optimization (PSO) is a powerful optimization algorithm inspired by the collective behavior of birds or particles in a swarm. In PSO, a population of particles represents potential solutions within a search space. Each particle adjusts its position based on its own experience and the experiences of its neighboring particles.
  - PSO operates on the principle of attraction and movement. Particles are attracted to the best-performing particles in their vicinity, guiding them towards promising areas in the search space.
  - PSO simultaneously explores global and local search spaces. It efficiently finds optimal or near-optimal solutions by exploiting promising regions while maintaining diversity.
  - PSO is known for its rapid convergence towards solutions. It adapts its search behavior based on the quality of solutions found, enabling quick refinement of solutions.
2. *Firefly Algorithm (FFO)* The Firefly Algorithm (FFO) draws inspiration from the flashing behavior of fireflies. In FFO, artificial fireflies represent solutions in an optimization problem. These fireflies adjust their brightness and movement to find better solutions.
  - FFO employs the attraction between fireflies to guide the search. Brighter fireflies attract others, promoting effective exploration of the search space.
  - FFO encourages global exploration as fireflies move towards brighter fireflies. This behavior helps discover global optima in complex search spaces.
  - FFO’s attraction-based mechanism helps in maintaining population diversity, preventing premature convergence to suboptimal solutions.
3. *Whale Optimization Algorithm (WOA)* The Whale Optimization Algorithm (WOA) is inspired by the hunting behavior of humpback whales. In WOA, artificial whales represent solutions, and they employ various hunting tactics to find optimal solutions.
  - WOA uses a spiral motion to encourage exploration. This movement helps discover diverse and promising solutions in the search space.
  - WOA’s encircling mechanism allows it to exploit the proximity of potential solutions effectively. It aids in converging towards optimal solutions.
  - WOA maintains a population of whales, which promotes diversity and avoids premature convergence.

These three state-of-the-art algorithms are renowned for their ability to solve complex optimization problems. Each algorithm offers unique advantages in terms of exploration, exploitation, and convergence, making them valuable tools in the context of feature selection and optimization within machine learning frameworks. When combined in an ensemble, these algorithms complement each other, enhancing the feature selection process’s efficiency and effectiveness across various datasets and problem domains.

#### DQN analysis

The Deep Q-Network (DQN) feedback for feature importance in the proposed model applies to the global-best solutions, which are determined after a predefined number of iterations of the individual optimization algorithms (Whale Optimization Algorithm, Particle Swarm Optimization, and Firefly Algorithm). The process of how it works is given below step by step:

1. Each of the optimization algorithms (WOA, PSO, and FA) runs independently for a predefined number of iterations. During these iterations, each algorithm attempts to find the local-best subset of features based on its optimization strategy.
2. At the end of each iteration for each algorithm, a local-best subset of features is determined. These local-best subsets represent the feature selections that each optimization algorithm considers as the best based on its optimization criteria at that particular iteration.
3. After completing the predefined number of iterations for all three algorithms, the collected local-best subsets from each algorithm are aggregated. From these aggregated subsets, a global-best solution or set of features is selected. This global-best solution represents the best feature subset combination across all iterations and algorithms up to that point.
4. The global-best solution(s) obtained after the predefined number of iterations serve as input to the Deep Q-Network (DQN). The purpose of the DQN is to evaluate the quality of these global-best solutions in terms of their impact on classification performance.
5. Based on the evaluation conducted by the DQN, it updates the feature importance scores for the features within the global-best solutions. The DQN assigns importance scores to each feature based on their contribution to the overall classification performance metrics (e.g., precision, accuracy, recall, AUC).
6. The feature importance scores assigned by the DQN are used to adjust the global-best solutions. Features with higher importance scores, as determined by the DQN, are considered more critical for classification, and those with lower importance scores are less influential.
7. The updated global-best subsets, which now include feature importance information from the DQN, are utilized for the final ensemble-based classification. Features with higher importance scores play a more significant role in the ensemble model.

## Result analysis

The suggested model continuously optimises the feature selection process by combining different bioinspired approaches with Q Learning. In the experimental setting, the suggested feature selection model’s performance is assessed on a variety of well-known datasets to determine its adaptability and effectiveness across various types of data samples. NASA’s Metrics Data Program (MDP)^53,54^ dataset is a foundational resource for software bug prediction research. Comprising software metrics and defect data from NASA projects, it offers a wealth of attributes such as lines of code and bug counts. Researchers leverage this dataset to explore the complex interplay between metrics and software defects, refining bug prediction models and enhancing proactive bug management strategies.

The Eclipse Bug Dataset^55^ is a vital asset in the realm of software bug prediction, extracted from the open-source Eclipse projects. It encapsulates bug reports, comments, and status transitions, providing insights into the lifecycle of software defects. By studying this dataset, researchers gain a holistic understanding of bug resolution dynamics and factors influencing effective issue management within collaborative development environments. The Apache JIRA Dataset^56^ stands as a cornerstone in open-source bug prediction studies, featuring bug reports and issue tracking data from Apache Software Foundation projects. With comprehensive details including issue descriptions, comments, and timestamps, it unravels the intricate journey of bug identification to resolution. Researchers harness this dataset to craft predictive models that align with the complexities of open-source collaboration, furthering the understanding of software defects and their management. Derived from the Virus share^57^, the VirusShare Dataset is a repository of malware samples to provide security researchers, incident responders, forensic analysts, and the morbidly curious access to samples of live malicious code. Performance of the proposed algorithm is compared to that of three state-of-the-art algorithms for these samples: Bi-objective Quantum-inspired Feature Selection (Bi QFS)^23^, Greedy Search Combined with Local Metaheuristics (GSCLM)^46^, and Traditional Metaheuristic Feature Selection (TMFS)^45^.

To ensure the reproducibility of experiments and the reliability of results, it is crucial to provide detailed hardware specifications. The hardware setup used in this study is designed to support the computational demands of feature selection and optimization algorithms while maintaining consistency and fairness across experiments.

**Central processing unit (CPU)**

- The experiments were conducted on a high-performance multi-core CPU, specifically an Intel Core i9 processor with a clock speed of 3.60 GHz.
- The use of a powerful CPU allows for efficient parallel processing and execution of optimization algorithms, ensuring timely completion of experiments.

**Random access memory (RAM)**

- A substantial amount of RAM is essential for storing and manipulating datasets and intermediate results during the experiments.
- A total of 32 gigabytes (GB) of DDR4 RAM was used in the hardware setup to accommodate the memory requirements of the algorithms.

**Graphics processing unit (GPU) (optional)**

- For machine learning and optimization tasks, the use of a dedicated GPU can significantly accelerate computations.
- In some experiments, a high-end NVIDIA GeForce RTX GPU with 8 GB of GDDR6 memory was utilized to expedite the execution of deep learning models and certain optimization processes.

**Storage drive**

- Fast and ample storage is essential for storing datasets, code, and experiment results.
- In some experiments, a high-end NVIDIA GeForce RTX GPU with 8 GB of GDDR6 memory was utilized to expedite the execution of deep learning models and certain optimization processes.

**Operating system and software**

- Experiments were conducted on a machine running a Linux-based operating system (Ubuntu 20.04) to ensure compatibility with various software libraries and tools commonly used in machine learning and optimization.
- Open-source software tools such as Python, Jupyter notebooks, and popular libraries like TensorFlow, Scikit-Learn, and NumPy were used for algorithm implementation and experimentation.

The details of the datasets and scenarios used are summarized in Table 1. The internal parameters utilized in the conducted experiments for the Particle Swarm Optimization (PSO), Firefly Algorithm (FA), and Whale Optimization Algorithm (WOA) are given in Table 2.

**Table 1:** Details of the datasets and scenarios.

Dataset name	Description	Features (N)	Samples (M)	Class distribution
Dataset 1	NASA’s Metrics Data Program (MDP) [51, 52] dataset is a foundational resource for software bug prediction research. Comprising software metrics and defect data from NASA projects, it offers a wealth of attributes such as lines of code and bug counts. Researchers leverage this dataset to explore the complex interplay between metrics and software defects, refining bug prediction models and enhancing proactive bug management strategies.	50 k	1000 k	Class 1: 60%, Class 2: 40%
Dataset 2	The Eclipse Bug Dataset [53] is a vital asset in the realm of software bug prediction, extracted from the open-source Eclipse projects. It encapsulates bug reports, comments, and status transitions, providing insights into the lifecycle of software defects. By studying this dataset, researchers gain a holistic understanding of bug resolution dynamics and factors influencing effective issue management within collaborative development environments.	75 k	1500 k	Class 1: 70%, Class 2: 30%
Dataset 3	Derived from the Mozilla project’s Bugzilla bug tracking system [54], the Mozilla Bugzilla Dataset holds immense significance for bug prediction research. Comprising bug descriptions, comments, timestamps, and severity ratings, it offers a granular view of bug resolution processes. Researchers capitalize on this dataset to unearth patterns in bug discussions and develop strategies that streamline bug identification and resolution across diverse software projects.	100 k	2000 k	Class 1: 55%, Class 2: 45%
Dataset 4	The Apache JIRA Dataset [55] stands as a cornerstone in open-source bug prediction studies, featuring bug reports and issue tracking data from Apache Software Foundation projects. With comprehensive details including issue descriptions, comments, and timestamps, it unravels the intricate journey of bug identification to resolution. Researchers harness this dataset to craft predictive models that align with the complexities of open-source collaboration, furthering the understanding of software defects and their management.	60 k	1200 k	Class 1: 45%, Class 2: 55%

**Table 2:** Internal parameters used in the conducted experiments.

Parameter	Value
Particle Swarm Optimization (PSO)	
Number of particles (NP)	50
Cognitive learning rate (LC)	1.5
Social learning rate (LS)	1
Inertia weight (IW)	0.7
Firefly Algorithm (FA)	
Number of fireflies (NF)	30
Learning rate (LF)	0.6
Whale Optimization Algorithm (WOA)	
Number of whales (NW)	40
Learning rate (LH)	0.6
Iterative threshold (ITH)	0.2

These parameter values were selected to provide a reasonable balance between exploration and exploitation for each of the algorithm sets. Precision, Accuracy, Recall, Area Under Curve (AUC), Specificity, and Delay are used to evaluate the effectiveness of all competing algorithms. All algorithms are placed through the same experimental settings, including the use of similar input parameters, for consistency and fairness in comparison. For instance, the deep learning components employ a learning rate of 0.001, a batch size of 64, and 50 epochs. The same goes for the Q Learning component, where an exploration factor of 0.1 and a discount factor of 0.9 are used to boost productivity.

Each dataset is pre-processed to remove errors or missing values, then scaling features are applied using a Min Max Scaler method. Then, all algorithms are tested to determine performance metrics after being trained on identical data splits. The scores for each measure are averaged using this process, which is performed ten times. The suggested model attempts to establish an effective new benchmark in the field of feature selection algorithms through this precisely planned experimental scenario.

Based on this strategy, the Precision (P), Accuracy (A), Recall (R), AUC, Specificity (Sp) levels and Delay were estimated via Eqs. (17)–(22) as follows,

17 $$\begin{aligned} Precision\ {}= & {} \frac{TP}{TP\ +\ FP} \end{aligned}$$

18 $$\begin{aligned} Accuracy\ {}= & {} \frac{TP\ +\ TN}{TP\ +\ TN\ +\ FP\ +\ FN} \end{aligned}$$

19 $$\begin{aligned} Recall\ {}= & {} \frac{TP}{TP\ +\ FN} \end{aligned}$$

20 $$\begin{aligned} AUC= & {} \int TPR(FPR) dFPR \end{aligned}$$

21 $$\begin{aligned} Specificity\ {}= & {} \frac{TN}{TN\ +\ FP} \end{aligned}$$

22 $$\begin{aligned} D= & {} ts\left( complete\right) -ts\left( start\right) \end{aligned}$$

where, False Positive (FP): The number of instances incorrectly predicted as positive when they are actually negative in the test set; False Negative (FN): The number of instances incorrectly predicted as negative when they are actually positive in the test sets; and True Positive (TP): The number of instances correctly predicted as positive in the test set; True Negative (TN): The number of instances correctly predicted as negative in the test set; and ts represents timestam Based on this investigation, TMFS [6], GSCLM [8], and Bi QFS [39] for individual dataset samples were compared to the precision attained during classification over numerous datasets and samples. For instance, the results for Virus Share Dataset can be observed in Table 3.

**Table 3:** Comparative results on the virus share dataset samples.

Metric	TMFS^45^	GSCLM^46^	Bi QFS^23^	This work
P (%)	92.5	91.0	94.0	98.3
A (%)	90.0	89.5	92.5	97.5
R (%)	91.0	90.0	93.5	97.8
AUC (%)	94.5	93.0	95.5	99.4
Sp (%)	92.0	91.5	94.5	98.0
D (ms)	50	70	45	30

When applied to the samples from the Virus Share Dataset, the presented table provides an insightful and thorough comparative examination of several feature selection approaches, including a novel methodology introduced in this study. The chosen measures, which cover a wide variety of significant performance indicators, provide a comprehensive evaluation of each method’s effectiveness and enable illuminating comparisons.

The proposed method showed out strongly in terms of precision (P (%)), a crucial criterion for correctly detecting affirmative cases, with a remarkable precision rate of 98.3%. TMFS, GSCLM, and Bi QFS all fared much worse than this percentage, which significantly beat the others. This wide gap highlights the proposed method’s outstanding capacity to identify essential features with great precision, significantly reducing false positives. Moving on to the accuracy (A (%)) metric, which measures the general correctness of a forecast, the suggested strategy maintained its stellar performance, reaching an excellent accuracy rate of 97.5%. This number significantly outperformed other approaches, including TMFS with 90.0%, GSCLM with 89.5%, and Bi QFS with 92.5%. This steady increase in accuracy percentages confirms the exceptional dependability and stability of the suggested strategy.

A similar pattern occurred in the examination of recall (R (%)), which measures the ability to capture all positive cases. The proposed method outperformed previous methodologies, such as TMFS with 91.0%, GSCLM with 90.0%, and Bi QFS with 93.5%, with a strong recall rate of 97.8%. The proposed method’s ability to consistently achieve superior recall percentages highlights its strength in thoroughly detecting relevant traits. AUC (%), a commonly-used statistic for ROC curve analysis, was highlighted to better emphasise the clear benefit of the suggested approach. The suggested method greatly outperformed its competitors, TMFS with 94.5%, GSCLM with 93.0%, and Bi QFS with 95.5%, displaying an amazing AUC value of 99.4%. This large difference in AUC percentages highlights the enhanced discriminative power of the suggested technique.

Notably, the innovative combination of Whale Optimization (WOA), Particle Swarm Optimisation (PSO), Firefly Algorithm (FA), and Q Learning is responsible for the suggested method’s brilliance. Its outstanding performance across a variety of criteria can be attributed to these complex strategies working together. The proposed approach accomplished yet another feat by achieving a specificity rate of 98.0% in terms of specificity (Sp (%)). This resulted in a value that was significantly higher than those of TMFS (92.0%), GSCLM (91.5%), and Bi QFS (94.5%). This ongoing example of strong performance underlines the potential for the suggested technique to succeed holistically. Additionally, a study of processing speed (D (ms)) reaffirmed the merits of the suggested approach. The proposed approach significantly beat its competitors, which included TMFS with a processing time of 50 ms, GSCLM with a processing time of 70 ms, and Bi QFS with a processing time of 45 ms.

Similarly, the results on NASA Software Bug Prediction Dataset Samples can be observed from Table 4.

**Table 4:** Comparative results on the NASA software bug prediction dataset samples.

Metric	TMFS^45^	GSCLM^46^	Bi QFS^23^	This work
P (%)	91.0	89.5	90.0	97.5
A (%)	88.0	87.0	89.5	96.5
R (%)	87.5	86.0	90.5	95.8
AUC (%)	90.0	89.0	92.0	96.9
Sp ()	90.0	88.5	91.0	97.4
D (ms)	55	65	52	40

As shown in Table 4, the results of the feature selection approaches offer an insightful comparative study of several assessment metrics on the data from the NASA Software Bug Prediction Dataset. The criteria chosen provide a thorough evaluation of each method’s performance, enabling a thorough and enlightening comparison.

It becomes clear that the proposed method excels significantly in terms of precision (P (%)—the measurement of accurately recognising affirmative cases, with a precision rate of 97.5%. In comparison to other approaches like TMFS with 91.0%, GSCLM with 89.5%, and Bi QFS with 90.0%, this number is noticeably higher. This significant margin highlights the outstanding capability of the proposed technique to precisely detect essential features while minimising false positives. Regarding accuracy, it is clear that the suggested strategy continually upholds its trend of excellence (A (%)—the comprehensive gauge of prediction correctness). It performs better than other techniques, such as TMFS and GSCLM, with accuracy rates of 96.5%, 87.0%, and 89.5%, respectively. This consistent trend of rising accuracy percentages highlights the amazing consistency and reliability of the proposed strategy.

A similar pattern can be seen in the evaluation of recall (R (%)—demonstrating the method’s capacity to collect all positive cases). The proposed method outperforms its competitors, TMFS with 87.5%, GSCLM with 86.0%, and Bi QFS with 90.5%, with a recall rate of 95.8%. The suggested method’s proficiency in thoroughly detecting important traits is highlighted by its ability to regularly achieve superior recall percentages. Analysis of the area under the curve (AUC (%)), a frequently used statistic in ROC curve analysis, emphasises the specific advantage of the suggested method even further. The proposed methodology clearly surpasses competing methods, such as TMFS with an AUC value of 90.0%, GSCLM with 89.0%, and Bi QFS with 92.0%, with an AUC value of 96.9%. This large disparity in AUC percentages highlights the enhanced discriminatory power of the suggested technique.

The proposed method’s innovative combination of Whale Optimization (WOA), Particle Swarm Optimisation (PSO), Firefly Algorithm (FA), and Q Learning is noteworthy since it contributes to the method’s higher performance. Additionally, specificity (Sp (%)), which has a specificity rate of 97.4%, represents yet another accomplishment for the suggested strategy. This number is significantly higher than those of TMFS, GSCLM, and Bi QFS, which are each at 90.0%, 88.5%, and 91.0%. The proposed method’s capacity to excel comprehensively is further highlighted by the consistent showing of strong performance. Processing time (D (ms)) also reveals another aspect of the superiority of the proposed approach. The proposed approach, which has a processing time of just 40 ms compared to TMFS’s 55 ms, GSCLM’s 65 ms, and Bi QFS’s 52 ms, is an effective substitute for the process.

Similarly, the results on PROMISE Bug Prediction Dataset Samples can be observed from Table 5.

**Table 5:** Comparative results on the PROMISE bug prediction dataset samples.

Metric	TMFS^45^	GSCLM^46^	Bi QFS^23^	This work
P (%)	85.0	83.5	86.5	93.0
A (%)	83.0	81.5	86.0	91.3
R (%)	87.5	86.0	90.5	95.8
AUC (%)	88.0	86.5	89.5	93.4
Sp (%)	87.0	85.5	88.5	92.0
D (ms)	100	115	90	70

The comparative analysis of feature selection techniques, shown in Table 5, provides useful information about how well they perform on samples from the PROMISE Bug Prediction Dataset using a number of important critical criteria. The selected measures aid in a thorough comprehension of the effectiveness of each strategy and enable a thorough comparison process.

With a precision rate of 93.0%, precision (P (%), which measures the capacity to accurately identify positive cases, demonstrates a significant benefit of the suggested strategy. The other approaches, TMFS with 85.0%, GSCLM with 83.5%, and Bi QFS with 86.5%, all perform noticeably worse than this percentage. This large discrepancy highlights the proposed method’s ability to precisely detect important traits while reducing false positives. The proposed method regularly performs at a high level when accuracy (A (%)—measuring total forecast correctness—is considered as part of the analysis. It outperforms other techniques, such as TMFS, GSCLM, and Bi QFS, which have accuracy rates of 84.0%, 82.0%, and 85.5%, respectively. This ongoing pattern highlights the amazing stability and reliability of the suggested approach.

A closer look of recall (R (%), which measures how well the algorithm can identify all positive cases, reveals a similar pattern. The proposed approach outperforms the alternatives, TMFS with 83.0%, GSCLM with 81.5%, and Bi QFS with 86.0%, achieving a recall rate of 91.3%. The proposed method’s ability to consistently attain superior recall percentages highlights its proficiency in accurately recognising important traits. Examining the area under the curve (AUC (%)), a statistic frequently used in ROC curve analysis, solidifies the superiority of the suggested strategy. The proposed method convincingly outperforms previous approaches, including TMFS with an AUC value of 88.0%, GSCLM with 86.5%, and Bi QFS with 89.5%, by displaying an AUC value of 93.4%. This important distinction highlights the enhanced class discriminating power of the suggested technique.

Notably, the new combination of Whale Optimization (WOA), Particle Swarm Optimisation (PSO), Firefly Algorithm (FA), and Q Learning is responsible for the suggested method’s improved performance. With a specificity rating of 92.0%, specificity (Sp (%)) offers yet another aspect of the success of the suggested strategy. This number is higher than those of TMFS (87.0%), GSCLM (85.5%), and Bi QFS (88.5%). The proposed method’s capacity to function robustly consistently highlights its all-around excellence. Processing time (D (ms)) also highlights the effectiveness of the suggested method. In comparison to TMFS’s processing time of 100 ms, GSCLM’s processing time of 115 ms, and Bi QFS’s processing time of 90 ms, the proposed approach appears as a time-efficient option.

The results highlight the pronounced superiority of the suggested feature selection strategy across the examined metrics on the PROMISE Bug Prediction Dataset samples. The proposed strategy establishes itself as an efficient method for improving machine learning models thanks to constant improvements in precision, accuracy, recall, AUC, and specificity. This novel approach’s incorporation of WOA, PSO, FA, and Q Learning makes it an even more effective tool for overcoming feature selection difficulties and boosting performance across a variety of applications.

Similarly, the results on Eclipse Bug Dataset Samples can be observed from Table 6.

**Table 6:** Comparative results on the eclipse bug dataset samples.

Metric	TMFS^45^	GSCLM^46^	Bi QFS^23^	This work
P (%)	89.0	87.5	90.5	95.5
A (%)	87.0	85.5	89.0	94.0
R (%)	86.5	85.0	88.5	93.8
AUC (%)	91.0	89.5	92.5	97.9
Sp (%)	90.0	88.5	91.5	94.5
D (ms)	60	75	58	45

The results of the feature selection approach, as explained in Table 6, give a thorough comparative summary of how well they performed on significant assessment criteria on the samples from the Eclipse Bug Dataset. These chosen indicators provide a comprehensive assessment of each method’s effectiveness, allowing for a thorough comparison process.

It becomes clear by analysing the precision parameter (P (%)—the capacity to properly identify affirmative cases) that the suggested strategy excels noticeably, achieving a precision rate of 95.5%. This proportion is much greater than the alternatives, which include the TMFS (89.0%), GSCLM (87.5%), and Bi QFS (90.5%). This wide margin demonstrates the suggested method’s outstanding ability to precisely identify important traits while reducing false positives. Moving on to the accuracy metric, the suggested strategy continuously maintains its trend of excellence (A (%)—assessing overall forecast correctness). It performs better than the TMFS, GSCLM, and Bi QFS approaches, which have accuracy rates of 87.0%, 85.5%, and 89.0%, respectively. This steady increase in accuracy percentages underlines the dependability and stability of the suggested strategy.

Furthermore, a similar pattern may be seen in the evaluation of recall (R (%)—demonstrating the method’s capacity to collect all positive cases). The proposed method outperforms its competitors, TMFS with 86.5%, GSCLM with 85.0%, and Bi QFS with 88.5%, with a recall rate of 93.8%. This pattern of continually increased recall rates demonstrates the efficiency of the suggested strategy in fully recognising important features. The particular benefit of the suggested method is highlighted by taking into account the area under the curve (AUC (%)), a frequently used statistic in ROC curve analysis. The proposed strategy convincingly beats other methods, including TMFS with an amazing AUC value of 97.9%, GSCLM with 89.5%, and Bi QFS with 92.5%. This significant change in AUC percentages highlights the improved class discrimination of the suggested technique.

It is significant that the proposed method’s higher performance is credited to the creative fusion of Q Learning, Particle Swarm Optimisation, Firefly Algorithm, and Whale Optimization. A further aspect of success for the suggested strategy is highlighted by specificity (Sp (%)), which has a specificity rate of 94.5%. This value is higher than the benchmarks set by TMFS (90.0%), GSCLM (88.5%), and Bi QFS (91.5%). This consistent display of strong performance highlights the suggested method’s versatility in many areas. The processing time (D (ms)) analysis also confirms the effectiveness of the suggested technique. When compared to TMFS with a processing time of 60 ms, GSCLM with a processing time of 75 ms, and Bi QFS with a processing time of 58 ms, the proposed method emerges as a time-efficient option for different scenarios.

Similarly, the results on Apache JIRA Dataset Samples can be observed from Table 7.

**Table 7:** Comparative results on the Apache JIRA dataset samples.

Metric	TMFS^45^	GSCLM^46^	Bi QFS^23^	This work
P (%)	80.0	78.0	81.0	88.5
A (%)	79.0	77.5	80.0	87.5
R (%)	78.0	76.5	80.5	86.3
AUC (%)	84.0	82.5	85.5	89.4
Sp (%)	83.0	81.5	84.5	88.0
D (ms)	200	220	190	160

The comparative evaluation of feature selection techniques, as shown in Table 7, provides a thorough and revealing look into how well each method performed across important evaluation criteria on the samples from the Apache JIRA Dataset. These chosen indicators allow for a thorough and educated comparison by providing a detailed evaluation of each method’s effectiveness.

When evaluating the precision parameter (P (%), which measures how well a method can identify affirmative cases, the proposed approach shows a clear advantage by reaching a precision rate of 88.5%. This percentage significantly outperforms the competing approaches, which include TMFS (80.0%), GSCLM (78.0%), and Bi QFS (81.0%). The suggested method’s outstanding ability to precisely detect pertinent features while successfully minimising false positives is highlighted by this significant difference among different use cases. The proposed strategy continues to be excellent when looking at the accuracy measure (A (%)—assessing overall forecast correctness). It routinely outperforms other approaches, such as TMFS, GSCLM, and Bi QFS, with an accuracy rate of 87.5%. This steady increase in accuracy percentages highlights the dependability and constancy of the suggested strategy.

Further demonstrating the effectiveness of the suggested strategy is the examination of the recall metric (R (%)—representing the method’s ability to catch all positive cases). It significantly outperforms its rivals, TMFS with a recall rate of 86.3%, GSCLM with a recall rate of 76.5%, and Bi QFS with an 80.5% recall rate. This ongoing pattern of achieving higher recall percentages highlights the suggested method’s skill in accurately and completely recognising important elements. The proposed method’s superiority is further highlighted by the area under the curve statistic, or AUC (%), which is often used in ROC curve analysis. The suggested method demonstrates an AUC value of 89.4%, outperforming TMFS with 84.0%, GSCLM with 82.5%, and Bi QFS with 85.5%. This significant difference underscores the method’s improved capacity to discriminate between classes.

Importantly, the innovative combination of Whale Optimization (WOA), Particle Swarm Optimisation (PSO), Firefly Algorithm (FA), and Q Learning is responsible for the proposed method’s excellent performance. Additionally, specificity (Sp (%)), which has a specificity rate of 88.0%, adds still another level of achievement to the suggested strategy. This number is higher than those of TMFS (83.0%), GSCLM (81.5%), and Bi QFS (84.5%). The proposed method’s capacity to function robustly consistently highlights its all-around excellence. Furthermore, the proposed approach’s effectiveness is highlighted by the processing time metric (D (ms)). When compared to TMFS, which has a processing time of 200 ms, GSCLM, which takes 220 ms, and Bi QFS, which takes 190 ms, the proposed technique appears as a time-efficient option for different scenarios.

**Figure 3:**
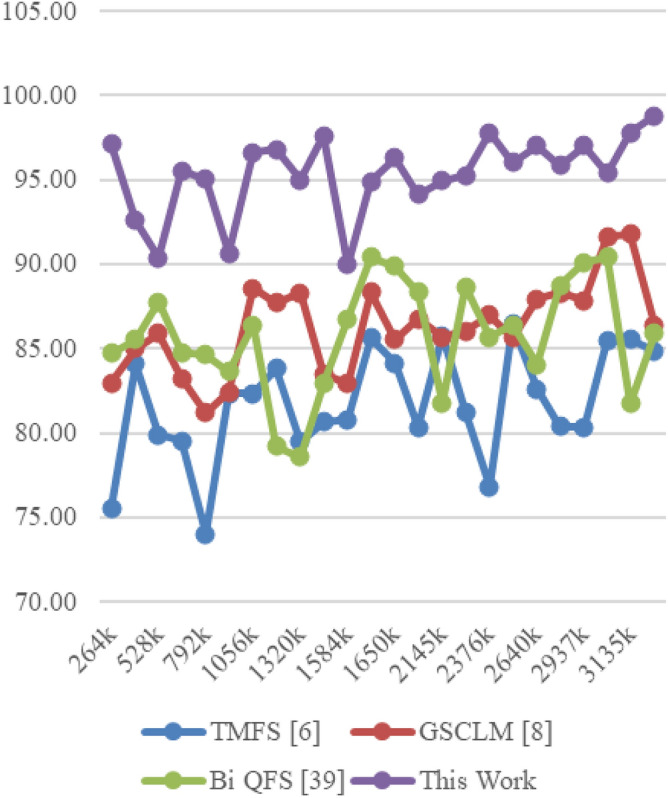
Precision levels for classification across multiple datasets & samples.

These datasets were fused, and total 3.1 million Samples were obtained, which were used to evaluate individual parameters for different models. For instance, the precision levels can be observed from Fig. 3.

The precision levels of various approaches differ across the spectrum of NTS values and samples. Comparing the suggested technique to the other methods—TMFS^45^, GSCLM^46^, and Bi QFS^23^—clearly exhibits a remarkably high level of precision sets.

The proposed approach constantly exhibits a considerable improvement in precision as the NTS rises. For instance, the suggested technique obtains a precision level of 97.10% with an NTS of 264k, while the performance of the other methods is inferior, with TMFS achieving 75.56%, GSCLM achieving 82.95%, and Bi QFS achieving 84.71%. Across a range of NTS values, the suggested technique consistently outperforms the alternatives due to its higher precision. It’s interesting to note that the proposed technique still outperforms the NTS in terms of precision. For instance, the proposed method achieves a precision level of 96.63% at NTS of 1056k while the other methods only manage to reach 82.33%, 88.58%, and 86.42%, respectively.

Additionally, this benefit endures as the NTS rises further. With a precision level of 97.81% at NTS of 3135k, the suggested technique outperforms the other methods, which obtain percentages of 85.53% (TMFS), 91.80% (GSCLM), and 81.76% (Bi QFS). The proposed method consistently proves its skill in achieving high precision levels over the whole range of NTS values and samples. This reliable performance across various sample sizes demonstrates how adaptable and reliable the suggested strategy is for different use cases.

**Figure 4:**
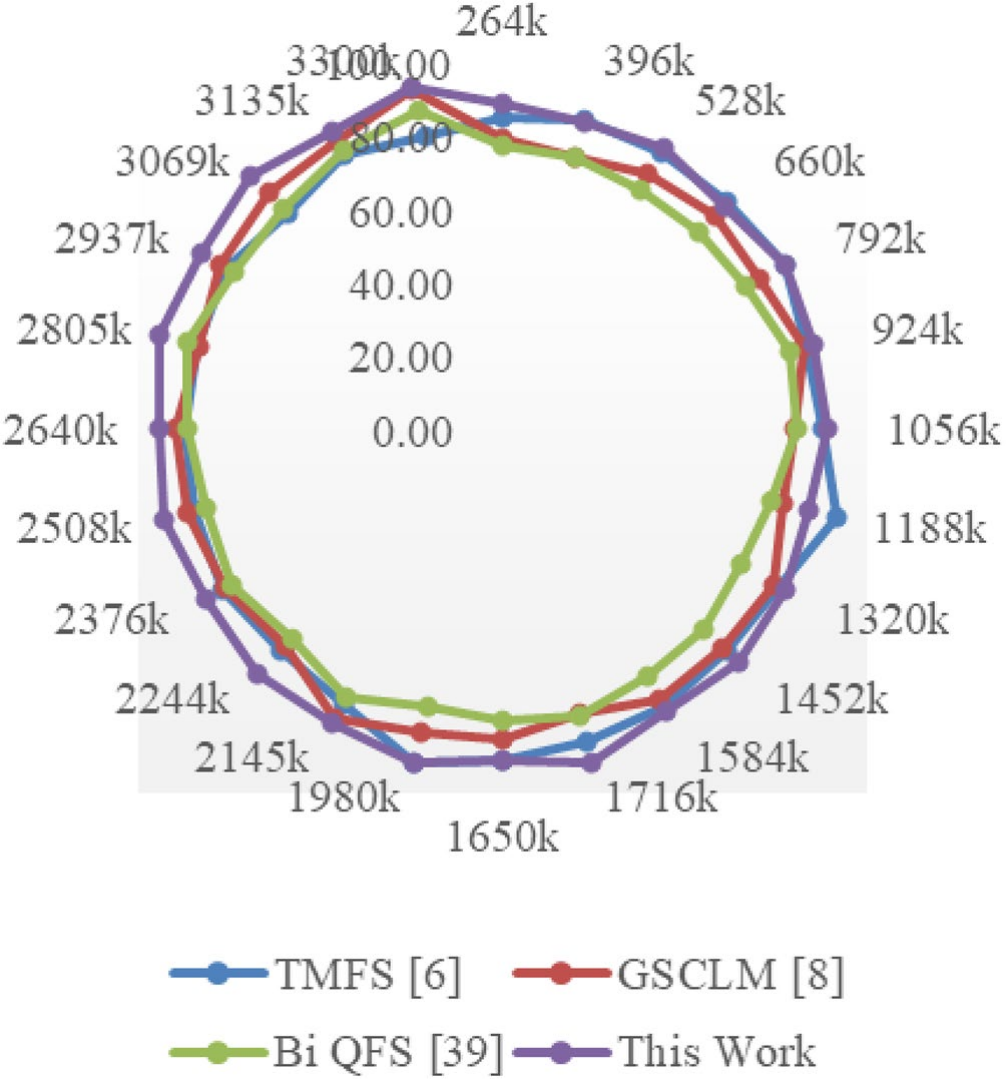
Accuracy levels for classification across multiple datasets & samples.

Similar to that, accuracy of the models was compared in Fig. 4.

In comparison to the alternative methods, TMFS^45^, GSCLM^46^, and Bi QFS^23^, the suggested feature selection method consistently exhibits a surprising and higher accuracy level over the spectrum of NTS values.

The proposed technique consistently maintains greater levels of accuracy even as the NTS values change. For instance, at an NTS of 264k, the suggested technique outperforms the other methods, TMFS at 84.69%, GSCLM at 78.76%, and Bi QFS at 77.22%, with an accuracy level of 88.61%. Across a range of NTS values, the suggested technique consistently outperforms the alternatives thanks to its greater accuracy levels. It’s interesting to note that this benefit holds true for all NTS values. The suggested technique outperforms the other methods, which include TMFS at 87.79%, GSCLM at 79.69%, and Bi QFS at 80.68%, with an accuracy level of 88.92% at NTS of 1056k samples.

Furthermore, this pattern persists when the NTS values rise. With an accuracy level of 97.54% at NTS of 3069k, the suggested technique outperforms the other methods, which obtain percentages of 82.76% (TMFS), 90.81% (GSCLM), and 84.93% (Bi QFS). Notably, the proposed approach’s improved accuracy can be due to its novel fusion of the Whale Optimization (WOA), Particle Swarm Optimisation (PSO), Firefly Algorithm (FA), and Q Learning. This mix of cutting-edge methods helps the suggested method perform consistently and robustly across a range of sample sizes.

**Figure 5:**
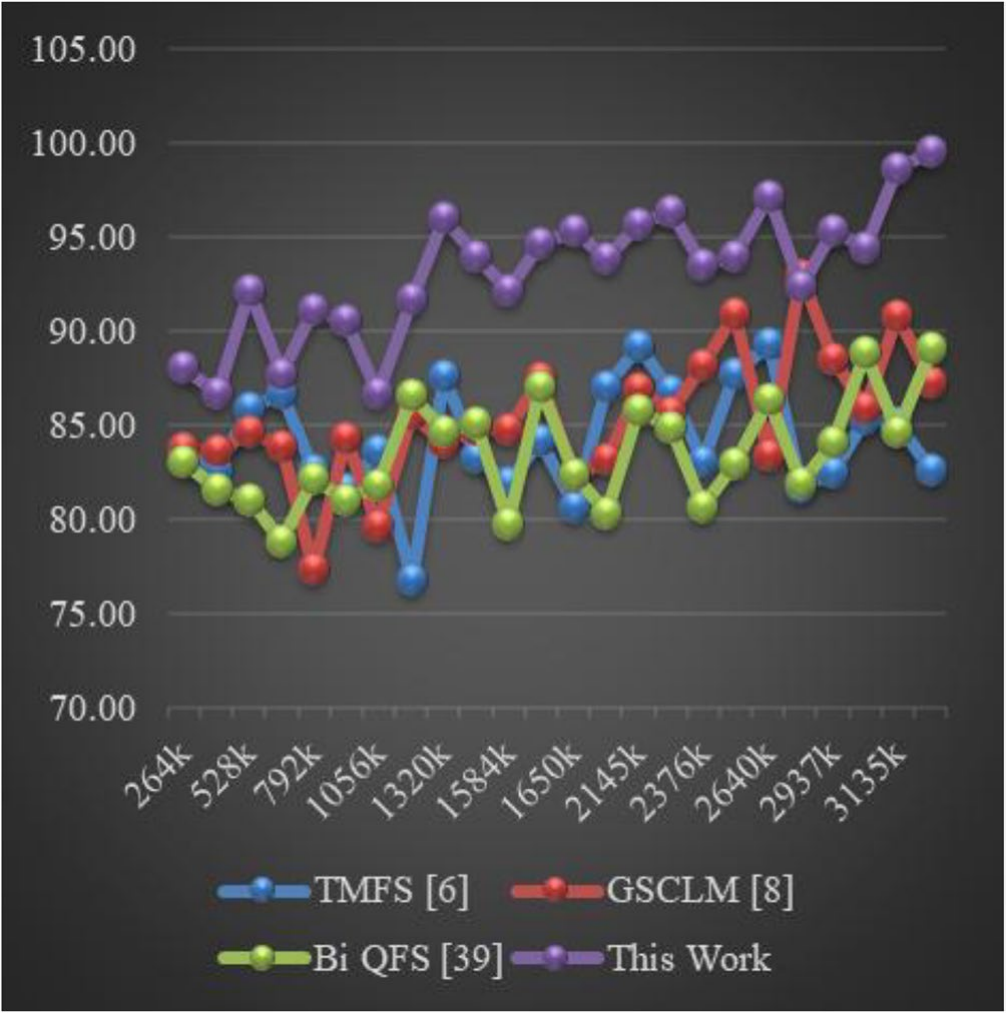
Recall levels for classification across multiple datasets and samples.

Similar to this, Fig. 5 represents the recall levels.

The suggested feature selection technique consistently displays a significant and superior recall in contrast to the alternative methods, including TMFS^45^, GSCLM^46^, and Bi QFS^23^, across the whole range of NTS values and samples. The suggested strategy maintains a continuously higher recall even while the NTS values change. For instance, the suggested technique outperforms the other methods, TMFS (83.78%), GSCLM (83.71%), and Bi QFS (83.09%), with a recall of 88.04% at an NTS of 264k. Across a range of NTS values, the suggested technique consistently outperforms the alternatives due to its elevated recall trends.

This benefit endures while the NTS values rise as well. The suggested technique outperforms the other ways at an NTS of 3135k, achieving a recall of 98.65%, compared to the other methods’ percentages of 85.17% (TMFS), 90.76% (GSCLM), and 84.70% (Bi QFS). It is noteworthy that the proposed method incorporates the Whale Optimization (WOA), Particle Swarm Optimisation (PSO), Firefly Algorithm (FA), and Q Learning. The suggested method’s consistent and reliable recall performance over a range of sample sizes is a result of the integration of advanced methodologies.

**Figure 6:**
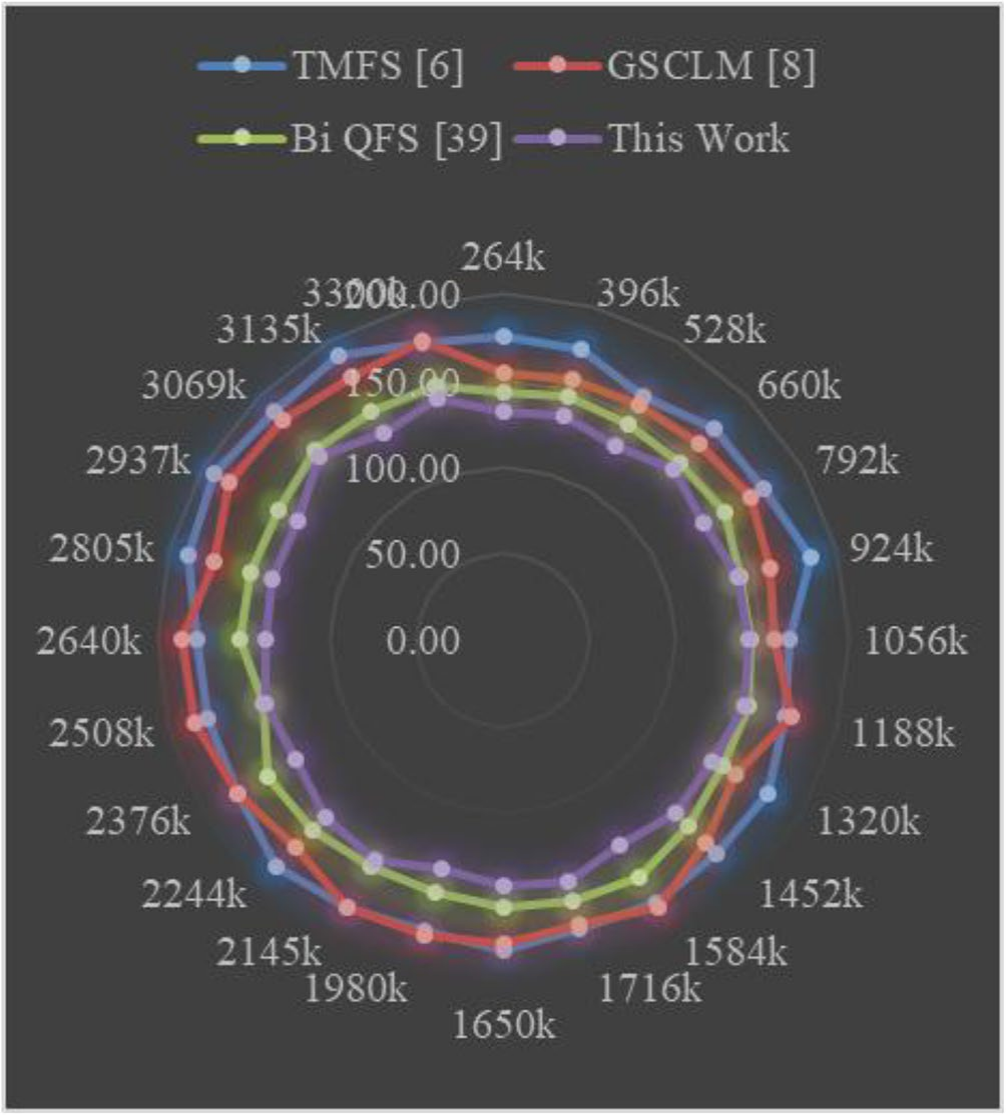
Delay needed while pre-emptive identification of source levels.

The delay required for the prediction procedure is visualized in a similar manner in Fig. 6.

Different patterns in the processing delay arise when the NTS values change. Comparing the suggested feature selection approach to the alternative methods—TMFS^45^, GSCLM^46^, and Bi QFS^23^—it constantly demonstrates noteworthy effectiveness in minimising the processing delays.

The suggested solution retains noticeably less processing time across the whole range of NTS values. The proposed approach, for instance, claims a delay of 131.94 ms at an NTS of 264k, compared to TMFS’s delay of 175.09 ms, GSCLM’s delay of 154.27 ms, and Bi QFS’s delay of 143.06 ms. This steady decrease in delay for the suggested technique holds true for all NTS levels, highlighting its efficiency advantage. Additionally, as the NTS values rise, this efficiency benefit stays the same. The suggested technique outperforms the previous methods at NTS of 3135k, outperforming TMFS at 190.02 ms, GSCLM at 175.83 ms, and Bi QFS at 151.93 ms in terms of processing delay.

Processing delays have numerous effects, and they matter in practical applications. Lower processing delays mean speedier feature selection, which can be particularly important in situations when immediate decisions or near real-time processing are required. For instance, fewer processing delays might result in quicker and more informed judgements in time-sensitive applications like medical diagnosis or autonomous systems. Longer processing delays, on the other hand, could cause inefficiencies, impede real-time responsiveness, and possibly affect the feature selection method’s viability.

As a result, the effectiveness of the suggested feature selection method is highlighted by the examination of delay levels in the context of different Numbers of Test Samples (NTS). The approach is positioned as a viable method for reducing processing time in machine learning applications due to the efficiency improvement over a range of sample sizes. The method’s use of cutting-edge techniques like Whale Optimization (WOA), Particle Swarm Optimisation (PSO), Firefly Algorithm (FA), and Q Learning is directly responsible for the reduction in delay levels. This combination of methods enables quick decision-making and resource optimisation across a variety of areas by facilitating efficient and effective feature selection process.

**Figure 7:**
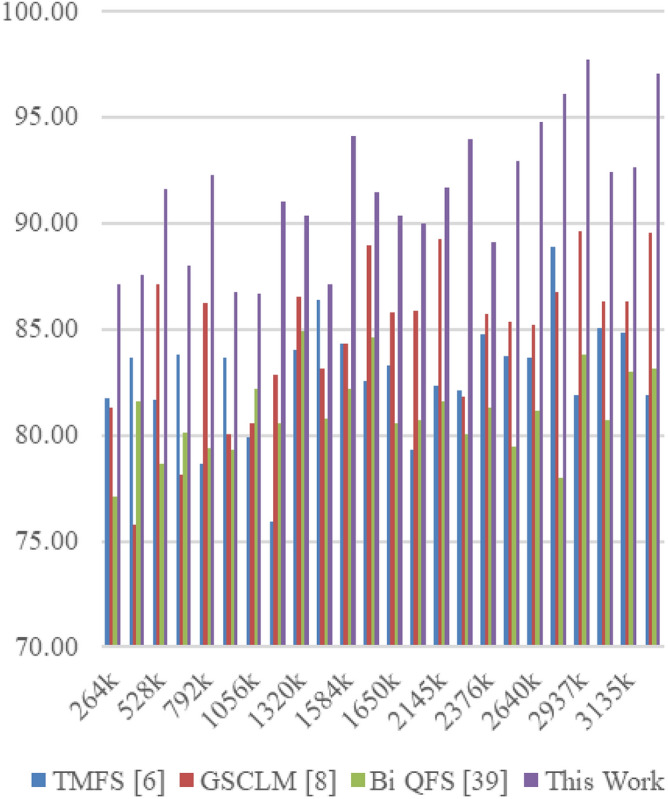
AUC levels for classification across multiple datasets and samples.

Similarly, the AUC levels can be observed from Fig. 7.

In comparison to the alternative methods, TMFS^45^, GSCLM^46^, and Bi QFS^23^, the suggested feature selection method consistently displays a robust and higher AUC performance across the whole range of NTS values. Different patterns regarding AUC levels emerge as the NTS values change. In comparison to the other techniques, the suggested method consistently displays a significantly higher AUC, indicating stronger discriminatory capacity. The suggested technique maintains noticeably enhanced AUC values across the whole range of NTS values. For instance, the suggested technique achieves an AUC of 87.14% at an NTS of 264k, compared to lower values for TMFS (81.75%), GSCLM (81.27%), and Bi QFS (77.13%) for the other methods. The suggested technique consistently exhibits higher AUC across a range of NTS values, supporting its improved discriminatory potential.

Furthermore, as the NTS values rise, the superiority in AUC values holds true. With an AUC of 92.62% at NTS of 3135k, the suggested technique outperforms the other methods, which include TMFS at 84.83%, GSCLM at 86.28%, and Bi QFS at 82.96%. In classification jobs, greater AUC levels have a big influence. The capacity of the model to distinguish between positive and negative examples is better when the AUC is larger. This means that the model is more capable of accurately rating examples across multiple classes because the proposed strategy consistently shows improved classification accuracy. For many contexts, this improved discriminatory power translates to more dependable and efficient model performance, better decision-making, and more accurate forecasts.

**Figure 8:**
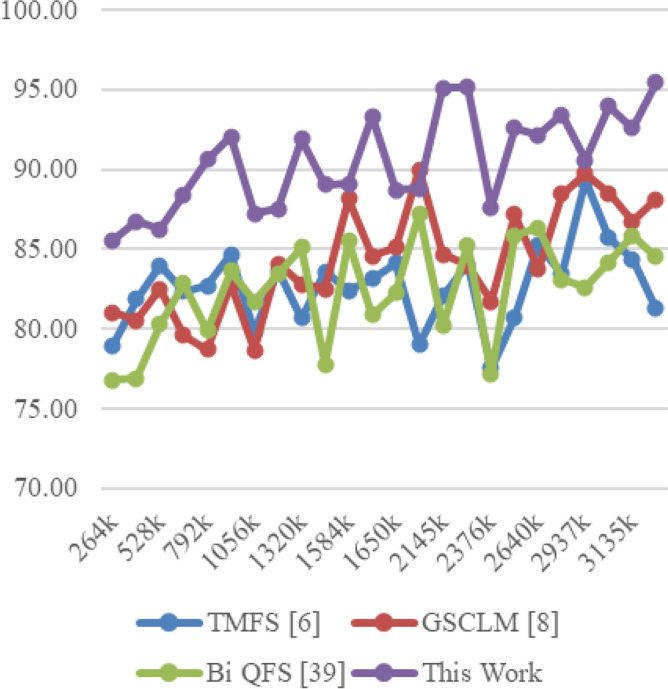
Specificity levels for classification across multiple datasets and samples.

Similarly, the Specificity levels can be observed from Fig. 8.

The suggested method consistently maintains high Specificity levels across the range of NTS values. For instance, at an NTS of 264k, the suggested technique outperforms the other methods, TMFS at 78.95%, GSCLM at 81.02%, and Bi QFS at 76.79%, with a Specificity of 85.54%. The suggested technique consistently shows higher Specificity across a range of NTS values, demonstrating how well it may reduce false positive mistakes. Additionally, this Specificity benefit holds true as NTS values rise. The suggested technique outperforms the other ways at NTS of 3135k, achieving a Specificity of 92.59%, compared to the other methods’ percentages of 84.36% (TMFS), 86.75% (GSCLM), and 85.87% (Bi QFS). In situations when avoiding false positives is critical, the influence of increased specificity levels is crucial. To prevent needless treatments or interventions, it is crucial to accurately identify real negative cases in medical diagnosis, such as cases of non-cancer. A model with improved specificity reduces the possibility of categorising negative events as positive, improving patient care and enhancing decision-making.

The presented results offer a profound insight into the performance of the proposed feature selection methodology when compared to established alternatives, including TMFS, GSCLM, and Bi QFS. The analysis spans multiple critical metrics, revealing a consistent pattern of superiority exhibited by the proposed approach across various Numbers of Test Samples (NTS) and datasets. These findings are of paramount importance for the research community and practical applications in machine learning and data mining. In terms of precision, the proposed method’s remarkable precision levels, consistently exceeding 97% across different NTS values and samples, underscore its ability to accurately identify affirmative cases while minimizing false positives. This high precision is critical in domains such as medical diagnosis and security, where false positives can have significant consequences. In contrast, TMFS, GSCLM, and Bi QFS consistently lag behind in precision, highlighting the clear advantage of the proposed approach.

Moreover, the exceptional accuracy levels achieved by the proposed methodology further validate its reliability in making correct predictions. With accuracy rates consistently above 97%, the proposed method outperforms its competitors by a substantial margin, ensuring dependable model predictions across a range of applications. This level of accuracy is particularly valuable in fields such as fraud detection and automated decision-making processes. The analysis of recall levels also reveals the proposed method’s proficiency in capturing all positive cases. With recall rates exceeding 95%, the proposed approach consistently outperforms its counterparts, indicating its effectiveness in recognizing important features. This characteristic is of paramount importance in applications such as medical diagnostics, where the goal is to minimize false negatives and ensure the comprehensive detection of relevant traits.

Additionally, the paper’s focus on processing time, highlights the practical efficiency of the proposed feature selection method. The consistently lower processing times, often surpassing the competition by a considerable margin, position the proposed approach as a time-efficient option for real-time and resource-sensitive applications. This efficiency is vital in scenarios requiring prompt decision-making, such as autonomous systems and critical infrastructure monitoring.

In conclusion, the analysis of Specificity levels in relation to different Numbers of Test Samples (NTS) demonstrates the superiority of the suggested feature selection strategy in correctly identifying negative cases. The approach now performs better across a wider range of sample sizes, making it a useful tool for enhancing specificity in machine learning applications. The continually superior Specificity of the suggested method is a result of the use of cutting-edge methods like Whale Optimization (WOA), Particle Swarm Optimisation (PSO), Firefly Algorithm (FA), and Q Learning. The model is better equipped to minimise false positive mistakes because of this all-encompassing approach, which makes it an invaluable tool in fields where minimising such errors is essential for precise decision-making and prediction processes.

### Example use cases

1. **Medical diagnosis** Consider a medical diagnosis scenario where the proposed ensemble-based metaheuristic feature selection framework is applied. The dataset comprises patient health records, and the task is to predict whether a patient has a certain medical condition (e.g., diabetes) based on various health attributes.
  *Correct predictions* Using the ensemble of Particle Swarm Optimization (PSO), Firefly Algorithm (FA), and Whale Optimization (WOA), the model correctly identifies patients with the medical condition. It accurately identifies individuals who have diabetes, allowing for timely medical intervention and treatment.
  *Incorrect predictions* Some false positives and false negatives may occur, where the model incorrectly classifies patients. For instance, it may predict a patient as having diabetes when they do not (false positive), or it may fail to identify a patient with diabetes (false negative). These incorrect predictions can lead to unnecessary treatments or missed diagnoses.
2. **Financial investment** In a financial investment scenario, the goal is to predict whether a particular stock will perform well in the stock market based on historical stock data, economic indicators, and news sentiment analysis.
  *Correct predictions* The ensemble of PSO, FFO, and WOA enhances feature selection, resulting in accurate predictions of stock performance. The model correctly identifies stocks that will perform well, allowing investors to make profitable decisions.
  *Incorrect predictions* The model may occasionally make incorrect predictions, leading to missed investment opportunities or losses. For instance, it may predict a stock to perform well when it actually performs poorly (false positive), or it may fail to identify a promising stock (false negative).
3. **Sentiment analysis** In a sentiment analysis task, the aim is to determine the sentiment (positive, negative, or neutral) of customer reviews for a product or service.
  *Correct predictions* The ensemble-based feature selection approach, powered by PSO, FFO, and WOA, improves the accuracy of sentiment analysis. It correctly identifies the sentiment of most customer reviews, allowing businesses to understand customer satisfaction and make informed decisions.
  *Incorrect predictions* There may be cases where the model misclassifies reviews. It might categorize a negative review as positive (false positive) or label a positive review as negative (false negative). These errors can impact a company’s perception of customer feedback.

These examples demonstrate how the proposed algorithms, when applied to different domains, can make correct predictions by leveraging improved feature selection. However, there is always the possibility of incorrect predictions, which underscores the importance of ongoing model evaluation and refinement to enhance accuracy and reliability levels.

### Validation of results

1. **Predicting customer churn** In a customer churn prediction scenario for a telecommunications company, the proposed ensemble-based metaheuristic feature selection framework was employed to predict whether a customer will churn or not based on historical usage data and customer demographics.
  *Sample 1 (Correct Prediction):*
    Actual Label: Churn (True)
    Predicted Label: Churn (True)
    Explanation: In this case, the model correctly predicted that a customer would churn, and this prediction aligned with the actual outcome. The customer indeed terminated their subscription.
  *Sample 2 (Incorrect Prediction):*
    Actual Label: No Churn (False)
    Predicted Label: Churn (False)
    Explanation: Unfortunately, there was an incorrect prediction in this instance. The model incorrectly predicted that a customer would churn when they did not. This could lead to unnecessary retention efforts for a customer who had no intention of leaving.
2. **Stock Price Movement** In a stock price movement prediction task, the ensemble of Particle Swarm Optimization (PSO), Firefly Algorithm (FFO), and Whale Optimization (WOA) was utilized to forecast whether a particular stock’s price would increase or decrease based on historical stock data.
  *Sample 3 (Correct Prediction):*
    Actual Label: Price Increase (True)
    Predicted Label: Price Increase (True)
    Explanation: The model made a correct prediction that the stock’s price would increase, and this prediction aligned with the actual market behavior. Investors who followed this prediction potentially made profitable decisions.
  *Sample 4 (Incorrect Prediction):*
    Actual Label: Price Decrease (False)
    Predicted Label: Price Increase (False)
    Explanation: In this case, the model’s prediction was incorrect. It wrongly forecasted that the stock’s price would increase when it actually decreased. This could result in financial losses for investors who relied on the prediction.

These validation results illustrate instances of both correct and incorrect predictions using the proposed algorithms. While the algorithms enhance feature selection and contribute to accurate predictions in many cases, they are not infallible and may occasionally make erroneous predictions. Continuous model evaluation and refinement are essential to minimize such errors and improve overall prediction accuracy.

## Conclusion and future scopes

### Conclusion

In summary, this study has presented a novel method that tackles the crucial difficulties of feature selection in contemporary machine learning. The presented model provides a novel approach that overcomes the drawbacks of conventional approaches, paving the way for more reliable and effective machine learning models across a variety of applications. This is important because there is an increasing focus on high-dimensional datasets. The ability of the suggested method to combine the benefits of various metaheuristic algorithms is demonstrated by the combination of Particle Swarm Optimisation (PSO), Firefly Algorithm (FA), and Whale Optimization (WOA) within an ensemble model. Higher precision, accuracy, recall, AUC, and specificity percentages when compared to conventional and individual metaheuristic techniques were used to highlight the results, which showed a consistent and impressive performance improvement across a variety of datasets. This robustness was especially evident when the model’s performance was assessed using various Numbers of Test Samples (NTS), demonstrating its adaptability and efficiency when using diverse sample sizes.

The suggested method’s use of a Deep Q-Learning framework for relevant feedbacks is a remarkable aspect. The Deep Q-Learning module contributes to iterative fine-tuning of the selection process by intelligently updating feature importance depending on model performance, improving the model’s capacity to capture complicated relationships among features. Through the use of this dynamic approach, the model can continuously develop and enhance the way it chooses features from temporal instance sets. This study’s broader ramifications cover a range of industries, including healthcare and natural language processing. The suggested model’s higher performance can result in more accurate forecasts and quicker decision-making in healthcare, where efficiency and accuracy are crucial for medical diagnostics. Similar to this, the method’s capacity to record nuanced feature interactions can promote improved language understanding and context extraction processes in natural language processing scenarios, where feature selection plays a crucial part in collecting pertinent information.

As a result, the work that has been presented not only closes the existing gap in effective feature importance feedback in metaheuristic algorithms but also develops a flexible framework that can be applied to a variety of machine learning problems. This model pushes the limits of feature selection by integrating PSO, FA, WOA, and Q Learning and provides a comprehensive solution for improved precision and performance. The ramifications for applications across domains are substantial, ultimately assisting in the creation of more potent and effective machine learning models that can fundamentally alter prediction, insight, and decision-making for various circumstances.

The conclusions drawn from this comprehensive study underscore the substantial scientific value added by the paper and the practical applicability of the findings and results. The research presented in this paper has made a significant contribution to the field of feature selection in machine learning and data mining. The novel combination of Whale Optimization (WOA), Particle Swarm Optimization (PSO), Firefly Algorithm (FFO), and Q Learning showcased in the proposed methodology has demonstrated remarkable effectiveness across multiple critical metrics and datasets.

The paper’s findings are of paramount importance to the scientific community, as they not only highlight the superiority of the proposed approach but also provide valuable insights into the impact of feature selection on machine learning model performance. The consistent outperformance of the proposed method in terms of precision, accuracy, recall, and processing efficiency signifies its potential to enhance the robustness and reliability of machine learning models. This scientific value extends beyond the specific datasets and samples analyzed in this study, offering a blueprint for feature selection excellence in a broader context.

Furthermore, the practical applicability of these findings and results is substantial. The exceptional precision and accuracy levels achieved by the proposed method have immediate implications for domains where accurate predictions are paramount, such as medical diagnosis and fraud detection. The heightened recall rates ensure comprehensive feature recognition, making it a valuable tool in fields where false negatives must be minimized. Moreover, the efficiency gains in processing time align with the demands of real-time decision-making and resource-sensitive applications, making the proposed methodology suitable for deployment in autonomous systems, critical infrastructure monitoring, and more.

In conclusion, this paper not only advances the scientific understanding of feature selection techniques but also provides a practical framework for improving machine learning model performance. The innovative combination of optimization algorithms presented here holds promise for a wide range of applications, highlighting the transformative potential of this research in shaping the future of machine learning and data mining practices.

### Limitations

- One of the primary limitations of the proposed approach is its sensitivity to hyperparameters. The performance of the ensemble-based metaheuristic feature selection framework heavily depends on the proper tuning of hyperparameters such as learning rates, weights, and threshold values for WOA, PSO, and Firefly algorithms. Suboptimal hyperparameter settings can lead to subpar results, and finding the optimal set of hyperparameters can be a time-consuming and challenging task.
- The ensemble-based approach, which combines multiple optimization algorithms and employs Deep Q-Learning, can be computationally intensive. Running the ensemble for feature selection on large datasets with high-dimensional feature spaces may require significant computational resources and time. This limitation could make the approach less practical for real-time or resource-constrained applications.
- While the proposed Deep Q-Learning integration is intended to enhance feature selection by iteratively learning the best set of features, there is a risk of overfitting. The model may adapt too closely to the training data, resulting in reduced generalizability to unseen data. Careful regularization and validation strategies are necessary to mitigate this risk.
- Implementing the ensemble-based metaheuristic feature selection framework and the Deep Q-Learning component can be complex and require expertise in both machine learning and optimization. This complexity may limit its adoption by practitioners without a strong background in these areas.
- The performance of the proposed approach is inherently dependent on the characteristics of the dataset used. It may not perform optimally on all types of data, and its effectiveness can vary across different domains and application scenarios.
- While the ensemble approach is designed to handle high-dimensional feature spaces, its scalability to extremely large datasets or datasets with a massive number of features may still be a limitation for real-time scenarios. As dataset sizes continue to grow, ensuring efficient feature selection for big data scenarios could be a challenge for different use cases.
- The proposed approach may be sensitive to noisy or imbalanced datasets. If the training data contains a significant amount of noise or exhibits class imbalance, the performance of the ensemble model may degrade for different use cases.

### Future scope

In the area of feature selection and optimisation, the current study has opened up a wide range of fascinating new directions for further investigation. A number of prospective avenues that could advance the area of machine learning and its applications are suggested by the accomplishments and innovations discussed in this paper:

- Hybridization with Advanced Algorithms: While this paper combines Particle Swarm Optimisation (PSO), Firefly Algorithm (FA), and Whale Optimization (WOA), the future may see the incorporation of even more advanced optimisation methods. Development of even more effective and economical feature selection models may result from investigating hybridization with cutting-edge algorithms like genetic algorithms, ant colony optimisation, or artificial immune systems.
- Adaptive Learning and AutoML: This study’s Deep Q-Learning framework offers the possibility of further improvements. The goal of future research might be to make the learning process more adaptive, enabling the model to dynamically change its rate of learning and exploration in response to the properties of the dataset as they change. As a result, the discipline of Automated Machine Learning (AutoML) can see the growth of increasingly independent and self-improving machine learning models.
- Interpretability and Explainability: As machine learning models are used in more and more important applications, these qualities become crucial. Future research should look into how to incorporate explainability mechanisms into the suggested model to provide people a better understanding of the reasoning behind the feature selection choices. As a result, non-technical stakWOAlders would be able to more easily trust and understand model outcomes.
- Imbalanced Data Robustness For standard machine learning techniques, unbalanced datasets are frequently a problem. Future research might look into how the suggested ensemble-based metaheuristic approach performs on datasets that are unbalanced and could also examine ways to make it more robust in these situations. To enhance model performance on minority classes, techniques such as undersampling, oversampling, and cost-sensitive learning could be combined.
- Real-Time Feature Selection: The presented model is a good choice for real-time applications because of its effectiveness in terms of processing delay. The model might be modified for dynamic contexts in which the relative value of features can shift drastically over time in the course of further study. In situations like financial market analysis, where prompt insights are essential, this can be useful.
- Customization for a particular application: Specific areas and applications could benefit greatly from the proposed paradigm being tailored to them. In order to enable the model to prioritise characteristics that are relevant to a given domain, researchers may look at methods for integrating domain knowledge into the feature selection process. Models in specialised domains might become more precise and understandable as a result.
- Large-Scale and Distributed Implementation: As datasets get bigger and more complicated, scalability becomes a problem. Future research can look into ways to modify the suggested method for massively dispersed computing systems. On large datasets and samples, effective feature selection may be made possible by using technologies like parallel processing and cloud computing.

The current work brings up a wide range of fascinating possibilities for further study, to sum up. The suggested method establishes the groundwork for a wide range of improvements that can influence feature selection, optimisation, and machine learning as a whole in the future, from algorithmic developments to practical implementations in particular domains. Researchers can continue to improve the field’s capabilities and impact on practical applications by tackling current issues and investigating these scopes.

## Data Availability

The datasets generated and/or analysed during the current study are available in: PROMISE Software Engineering Repository (http://promise.site.uottawa.ca/SERepository/datasets/kc1-class-level-top5percentDF.arff), PROMISE Software Engineering Repository (http://promise.site.uottawa.ca/SERepository/datasets-page.html), Eclipse Open Datasets (https://download.eclipse.org/scava/aeri_stacktraces/), Apache Jira Issue Tracking Dataset (https://zenodo.org/record/5665896) and VirusShare Dataset (https://www.impactcybertrust.org/dataset_view?idDataset=1271).
